# Super-enhancer-associated TMEM44-AS1 aggravated glioma progression by forming a positive feedback loop with Myc

**DOI:** 10.1186/s13046-021-02129-9

**Published:** 2021-10-25

**Authors:** Erbao Bian, Xueran Chen, Li Cheng, Meng Cheng, Zhigang Chen, Xiaoyu Yue, Zhengwei Zhang, Jie Chen, Libo Sun, Kebing Huang, Cheng Huang, Zhiyou Fang, Bing Zhao, Jun Li

**Affiliations:** 1 Department of Neurosurgery, The Second Affiliated Hospital of Anhui Medical University, Hefei, 230601 China; 2 Cerebral Vascular Disease Research Center, Anhui Medical University, Hefei, 230601 China; 3 Department of Laboratory Medicine, Hefei Cancer Hospital, Chinese Academy of Sciences, No. 350, Shushan Hu Road, Hefei, 230031 Anhui China; 4 Anhui Province Key Laboratory of Medical Physics and Technology; Institute of Health and Medical Technology, Hefei Institutes of Physical Science, Chinese Academy of Sciences, No. 350, Shushan Hu Road, Hefei, 230031 Anhui China; 5 School of pharmacy, Anhui Medical University, Hefei, 230032 China

**Keywords:** Glioma, lncRNA, TMEM44-AS1, Super-enhancer, Myc

## Abstract

**Background:**

Long non-coding RNAs (lncRNAs) have been considered as one type of gene expression regulator for cancer development, but it is not clear how these are regulated. This study aimed to identify a specific lncRNA that promotes glioma progression.

**Methods:**

RNA sequencing (RNA-seq) and quantitative real-time PCR were performed to screen differentially expressed genes. CCK-8, transwell migration, invasion assays, and a mouse xenograft model were performed to determine the functions of TMEM44-AS1. Co-IP, ChIP, Dual-luciferase reporter assays, RNA pulldown, and RNA immunoprecipitation assays were performed to study the molecular mechanism of TMEM44-AS1 and the downstream target.

**Results:**

We identified a novel lncRNA TMEM44-AS1, which was aberrantly expressed in glioma tissues, and that increased TMEM44-AS1 expression was correlated with malignant progression and poor survival for patients with glioma. Expression of TMEM44-AS1 increased the proliferation, colony formation, migration, and invasion of glioma cells. Knockdown of TMEM44-AS1 in glioma cells reduced cell proliferation, colony formation, migration and invasion, and tumor growth in a nude mouse xenograft model. Mechanistically, TMEM44-AS1 is directly bound to the SerpinB3, and sequentially activated Myc and EGR1/IL-6 signaling; Myc transcriptionally induced TMEM44-AS1 and directly bound to the promoter and super-enhancer of TMEM44-AS1, thus forming a positive feedback loop with TMEM44-AS. Further studies demonstrated that Myc interacts with MED1 regulates the super-enhancer of TMEM44-AS1. More importantly, a novel small-molecule Myc inhibitor, Myci975, alleviated TMEM44-AS1-promoted the growth of glioma cells.

**Conclusions:**

Our study implicates a crucial role of the TMEM44-AS1-Myc axis in glioma progression and provides a possible anti-glioma therapeutic agent.

**Supplementary Information:**

The online version contains supplementary material available at 10.1186/s13046-021-02129-9.

## Background

Gliomas are the most frequent primary tumor of the central nervous system, accounting for approximately 45% of all intracranial tumors [1]. The World Health Organization distinguishes four grades of glioma (I, II, III, IV) according to their predicted clinical behavior and morphological characters [2]. Although intense therapeutic efforts include surgery followed by combined radiation and chemotherapy, glioblastoma (GBM) is a high-grade glioma (WHO grade IV), with a median survival rate of approximately 14 months [3]. This unfavorable prognosis reflects the biological properties considered as hallmarks of cancer, such as proliferative signaling and activating invasion of glioma cells [4]. Hence, a novel therapeutic strategy based on a deep understanding of the regulatory mechanisms underlying glioma progression is urgently needed.

Long non-coding RNA (lncRNA) is a type of RNA transcript that is larger than 200 nucleotides and has weak coding potential. LncRNAs have been reported to participate in a broad scope of crucial biological functions, and their dysregulation results in disease states, including malignancy [5]. Mechanistically, lncRNAs exert their efficient and effective actions mainly by interaction with proteins/RNAs, transcriptional regulation, epigenetic regulation, and mRNA stabilization and signaling pathway [6]. In addition, the clusters of active enhancers known as super-enhancers (SEs) are involved in the dysregulated expression of lncRNA in several types of tumors. Super-enhancers (also known as stretched or clustered enhancers) bind with a high density of transcription factors and coactivators, providing cooperative binding and synergistic gene activation [7]. Super-enhancers are enriched in cell-type-specific genes that define cell identity and differentiation [8]. Importantly, super-enhancers frequently drive high-level expression of lncRNAs with tumor-promoting functions [9]. Thus, there is currently great interest in characterizing super-enhancers that drive candidate oncogenic lncRNA in glioma pathogenesis. Interestingly, lncRNAs have been reported to be transcript units between protein-coding genes, and they exhibit highly tissue- and cell-specific expression patterns in cancer cells [10]. However, the biological function of specific transcription dysregulated lncRNAs in glioma and the underlying mechanisms of their transcriptional activation are still largely unknown.

Myc, a bHLHZip transcription factor, is implicated in regulating many cellular processes, including cell differentiation, growth, proliferation, and programmed cell death [11]. A previous study showed that Myc expression was associated with glioma grade and found high Myc expression in approximately 60–80% of GBM [12]. In addition, the astrocyte lineage mice of genetically modified Myc can sufficiently induce the formation of gliomas resembling the human disease [13]. Others have proposed that Myc functional mutations promote glioma progression, suggesting targeting mutant Myc may be a better strategy for the treatment of glioma [14]. A recent study found that Myc binds to enhancers and promoters to activate the expression of target genes in a cell-type-specific manner [15, 16]. However, there has been no report about SE-associated lncRNA targeted by Myc and how the transcriptional regulation of Myc on SE has not been studied. We demonstrated that the upregulation of TMEM44-AS1 activated by Myc and the effect of TMEM44-AS1 on downstream targets Myc and EGR1/IL-6 is dependent on SerpinB3, and the interaction of Myc with MED1 bound to the TMEM44-AS1 SE and promoted its glioma-specific transcription activation in glioma progression.

## Materials and methods

### Tissue samples

Tissues samples from glioma patients who underwent surgical resection at the Department of Neurosurgery of The Second Affiliated Hospital of Anhui Medical University (Hefei, China) after obtaining the patient’s informed consent. All cases included 46 glioma samples and 13 normal samples were directly preserved in stored at − 80 °C. Tumor samples were pathologically graded as 19 low-grade tumors (LGG) and 27 GBM based on the WHO criteria. The primary tumor characteristics and clinical information was shown in Supplementary Table 1. The present study was approved by the Research Ethics Committee of The Second Affiliated Hospital of Anhui Medical University.

### Cell culture and treatment

U251, T98G, LN-18, and A172 glioma cell lines were obtained from American Type Culture Collection (Manassas, VA). (H4, SF126, and U87) and normal human astrocytes (NHAs) were purchased from The Cell Bank of the Chinese Academy of Sciences and (Shanghai, China) and Sun Yat-Sen University, respectively. The patient-derived glioblastoma cells (PN12, PN16, MES23, and MES27) were isolated from surgical specimens as reported in our previous study [17]. The cell lines were regularly checked for Mycoplasma contamination to guarantee that experiments were performed only with Mycoplasma-free cells. Myci975 was purchase from MedChemExpress (Princeton, NJ). Human-specific small interfering RNA (siRNA) for TMEM44-AS1, EGR, Myc, MED1, and negative control were transfected into the glioma cells via the DharmaFECT transfection reagent (Thermo Scientific Dharmacon) according to the instructions in the manufacturer’s manual. All the siRNAs were purchased from Genepharma Technology (Shanghai, China). We cloned the cDNA of TMEM44-AS1 into the pcDNA3.1 vector (Invitrogen) and verified it by sequencing. The plasmids and empty plasmids were transfected with the FuGENE transfection reagent (Life Technologies, USA). SiRNA target sequences were listed in Supplementary Table 2.

### In vitro functional assays

**Cell proliferation assay** Glioma Cells were seeded into a 96-well plate at a density of 3000 cells per well in triplicate and incubated at 37 °C 4 days post-infection. The cell number was quantified using Z1 Particle Counter (Beckman Coulter). The cell proliferation curve was generated by the OD measured at 490 nm.

**Colony formation assay** 400 glioma cells were planted into the 6-well plate in triplicate, continually maintained in the complete medium for 14 days. Following incubation, cells were washed three times with PBS and fixed with 4% neutral paraformaldehyde solution at room temperature for 30 min. Cell colonies formed were stained with Giemsa for 15 min.

**Cell migration and invasion assay** To measure glioma cell migration, 8-mm pore size culture inserts (Transwell; Costar) were placed into the wells of 24-well culture plates, the upper and the lower chambers were separated. The lower chamber was filled with 500 μl culture medium supplemented with 10% FBS. Cells at a concentration of 5 × 10^4^ cells in 500 ul of serum-free medium were inoculated in the upper chamber, coated with (invasion assay) or without (migration assay) growth factor reduced Matrigel, while medium containing medium in the lower chamber. Fixed cells were dyed with 0.1% crystal violet for 20 min and counted by microscopy (Zeiss).

### RNA-FISH and subcellular fraction

The RNA FISH kit (Life Technologies) was applied to analyze TMEM44-AS1 subcellular localizations in glioma cells, The RNA FISH probe mix for TMEM44-AS1 was synthesized by RiboBio (Guangzhou, China), and the assay was performed with the kit according to the manufacture’s protocol. The levels of GAPDH (cytoplasm control) were measured, U6 (nucleus control), respectively.

### Dual-luciferase reporter assays

The indicated regions of TMEM44-AS1 promoter and super-enhancer were directly inserted into pGL3-promoter and pGL3-enhancer plasmid, respectively. The pcDNA3.1-Myc or vector was co-transfected into glioma cells. The pGL3 vector was utilized as a negative control. The Dual-Luciferase Reporter Assay System (Promega, USA) was used to detect luciferase activity, and the firefly luciferase activity was normalized to Renilla luciferase values.

### Chromatin immunoprecipitation (ChIP)

ChIP analysis was carried out following the manufacturer’s instructions for SimpleChIP Plus Sonication Chromatin IP Kit (Cell Signaling Technology). Briefly, the chromatin of the glioma cells was treated with 2 μg anti-H3K27ac, anti-Myc, anti-EGR1 (Cell Signaling Technology), or MED1 (Sigma). Immunoprecipitated DNA was detected through RT-qPCR using specific primers (Supplementary Table 3).

### RT-qPCR, western blot, immunocytochemistry, and immunofluorescence

Immunocytochemistry, immunofluorescence, RT-qPCR, and western blot were done according to our standard protocol, as described previously [38]. Primers were listed in Supplementary Table 4. Antibodies used were anti-MED1, anti-EGR1, anti-PCNA (Abcam), anti-Serpin3B (Proteintech), or anti-Myc, anti-Ki67 (Cell Signaling Technology).

### Co-immunoprecipitation (co-IP) assay

Co-immunoprecipitation (Co-IP) was used to detect the interaction between Myc (Abcam) and MED1(Santa Cruz Biotechnology), and antibody-protein complex capture with Protein A/G agarose (Bimake, B23201). Then, the obtained complexes were subjected to western blot analysis with the following anti-Myc or anti-MED1.

### Tumor formation study in vivo

A xenograft model of BALB/c nude mice was established to elucidate the impacts of TMEM44-AS1 on tumor formation in vivo. 4-week-old BALB/c-nude mice were obtained from Beijing HFK Bio-Technology Co., Ltd. (China). All mouse experiments were performed based on the approval of the Animal Research Committee of Anhui Medical University. Animals were maintained and handled following the recommendations of the guidelines for Institutional and Animal Care and Use Committees. A total of 20 mice were randomly divided into two groups (*n* = 10 per group). Infected glioma (5 × 10^6^ cells/mouse) suspended in 100 μl PBS were inoculated into the subcutaneous region of nude mice. Tumor size was monitored every seven days by measuring the length and width with calipers, and tumor volumes were calculated with the formula: V = 0.5 × L (length) × W^2^ (width). Mice were sacrificed on day 42 after glioma cell inoculation. Tumor volume and weight of glioma tissues were monitored over time as indicated.

### RNA-seq analysis

Total RNAs from glioma cells transfected by shTMEM44-AS1 were harvested and sent to a commercial company for transcriptome sequencing. Raw reads were trimmed to remove low-quality bases and adaptor sequences using fastp (default parameters). Reads were mapped to the hg38 genome using HISTAT2. After generating the BAM file, then featureCounts to quantify read counts of each gene. Reads per kilobase per million (RPKM) values based on gene counts were calculated by using in house scripts in R.

### ChIP-seq analysis

The raw fastq file (GSE36354) from the European Nucleotide Archive was download by using Aspera, and reads are then mapped to the reference using Bowtie2, and Samtools was used for sorting and indexing. BigWig files were created from bam files for each sample using genomeCoverageBed and bedGraphToBigWig and the peak calling by using macs14. The Super-Enhancers (Stitching distance: 12.5 kb) were identified by the ROSE, and the downstream analysis using deepTools and visually inspected with IGV.

### RNA pulldown + MS/WB

RNA pull-down assay was performed using the BersinBioTM RNA pulldown Kit (BersinBio, Guangzhou, China) according to the manufacturer’s instruction. Sense and antisense strands of TMEM44-AS1 cDNA were synthesized by Sangon Biotech (Shanghai, China). Biotin-labeled TMEM44-AS1 were obtained using in vitro transcription with T7 RNA polymerase and Biotin RNA Labeling Mix (Roche) and followed by incubation with extracts separated from glioma cells. The pulldown fraction was separated using SDS-PAGE and visualized by silver-stained. Finally, the channel subtype composition was verified by mass spectrometry (MS) or western blotting.

### RNA immunoprecipitation (RIP) assay

The RIP experiment was processed following the protocol of the Magna RIP RNA-Binding Protein Immunoprecipitation Kit (Millipore). Cells (1 × 10^7^) were harvested and resuspended with RIP lysis buffer, followed by a step of centrifugation to remove the cell debris. The beads-antibody complex was then mixed with RIP immunoprecipitation buffer and cell lysis at 4 °C overnight. The RNA-protein complexes were isolated, followed by proteinase K digestion, and the coprecipitated RNAs were used for cDNA synthesis and evaluated by RT-qPCR.

### Copy number data analysis

The gene expressions of glioma samples and Copy-number data were retrieved from the Xena dataset (https://xenabrowser.net/). Gene expression data were plotted against the copy number, and the Pearson correlation was performed to determine the correlation coefficient and the *p*-value.

### Statistical analysis

Data were expressed as the mean ± SD of the results of three independent experiments in which each assay was performed in triplicate. Statistical analysis was performed with Student’s t-test or one-way ANOVA followed by Student’s t-test. The relationship was analyzed with Pearson^’^s correlation. P<0.05 is considered a significant difference.

## Results

### TMEM44-AS1 expression was significantly up-regulated in glioma and associated with prognosis

**Fig. 1:**
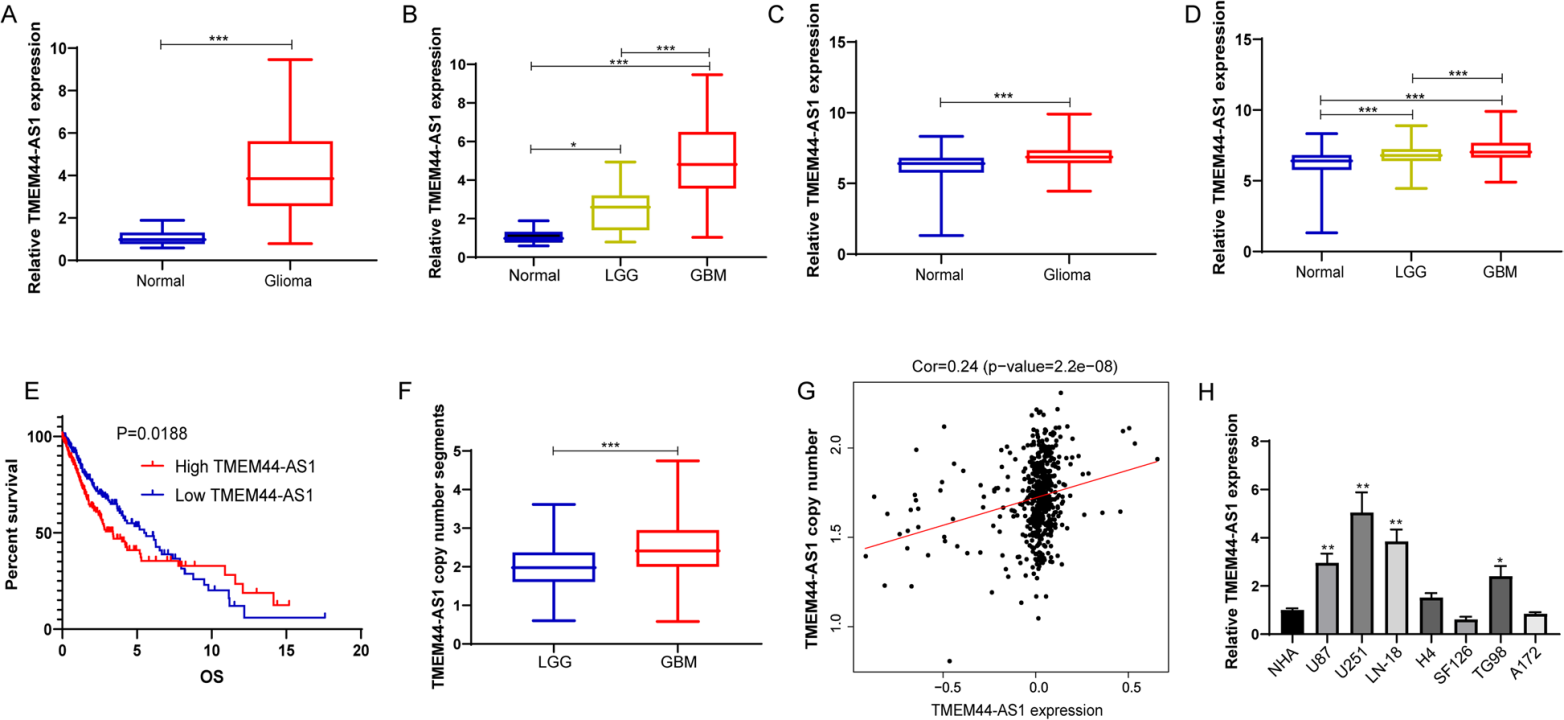
Expression of TMEM44-AS1 in various tissues and cells. (**A**) Relative TMEM44-AS1 expression in glioma and normal brain tissues from our cohort. (***P < 0.001). (**B**) The TMEM44-AS1 expression is shown according to the histopathologic grades. (*P < 0.05, ***P < 0.001). (**C**) Relative TMEM44-AS1 expression in glioma and normal brain tissues from GTEX(*n* = 1136) and the TCGA database(*n* = 689). (***P < 0.001). (**D**) The TMEM44-AS1 expression is shown according to the histopathologic grades of TCGA gliomas. LGG (*n* = 523); GBM (*n* = 166). (***P < 0.001). (**E**) Kaplan-Meier curves of overall survival time of patients based on TMEM44-AS1 expression in glioma samples obtained from the TCGA database. (**F**) TMEM44-AS1 copy number segments in LGG and GBM were tested using the TCGA database. (***P < 0.001). (**G**) The correlation between TMEM44-AS1 copy number and TMEM44-AS1 expression in glioma from the TCGA database (*n* = 528). (H) Relative TMEM44-AS1 in NHA, U87, U251, LN-18, H4, SF126, T98G, A172 cell lines (*P < 0.05, **P < 0.01)

To investigate the role of TMEM44-AS1 in glioma, we observed TMEM44-AS1 expression in glioma tissue versus normal brain tissue from our cohort. As shown in Fig.1A, compared with the normal brain tissue group, expression of TMEM44-AS1 was significantly increased in glioma tissue. To explore whether the upregulation of TMEM44-AS1 is correlated to the pathogenesis of glioma, TMEM44-AS1 expression was observed in different histopathologic grades of malignant glioma. A significant increase in TMEM44-AS1 expression was observed in GBM tissues, whereas a slight increase in LGG tissues (Fig.1B). Similarly, high expression of TMEM44-AS1 in glioma tissue versus normal brain tissues was confirmed from integrated analyses of the TCGA and GTEx databases (Fig.1C). Additionally, high expression of TMEM44-AS1 in glioma was correlated with pathogenesis grade, is closely associated with poor prognosis in glioma (Fig.1D-E).

Next, we sought to delineate the molecular mechanism underlying the up-regulation of TMEM44-AS1 in glioma. Genomic gain or amplifications associated with the activation of proto-oncogenes is an essential mechanism in human glioma [18]. To evaluate whether differences in the expression of TMEM44-AS1 were due to amplification of this locus, we analyzed the copy number of TMEM44-AS1 in glioma samples. Interestingly, the copy number of TMEM44-AS1 was frequently increased and positively correlated with TMEM44-AS1 expression in human glioma samples (Fig.1F-G). Additionally, TMEM44-AS1 expression was measured in normal human astrocytes (NHA) and a panel of glioma cell lines. The expression of TMEM44-AS1 was upregulated in glioma cell lines compared to the NHA (Fig.1H).

### TMEM44-AS1 promotes glioma cell proliferation, migration, and invasion

**Fig. 2:**
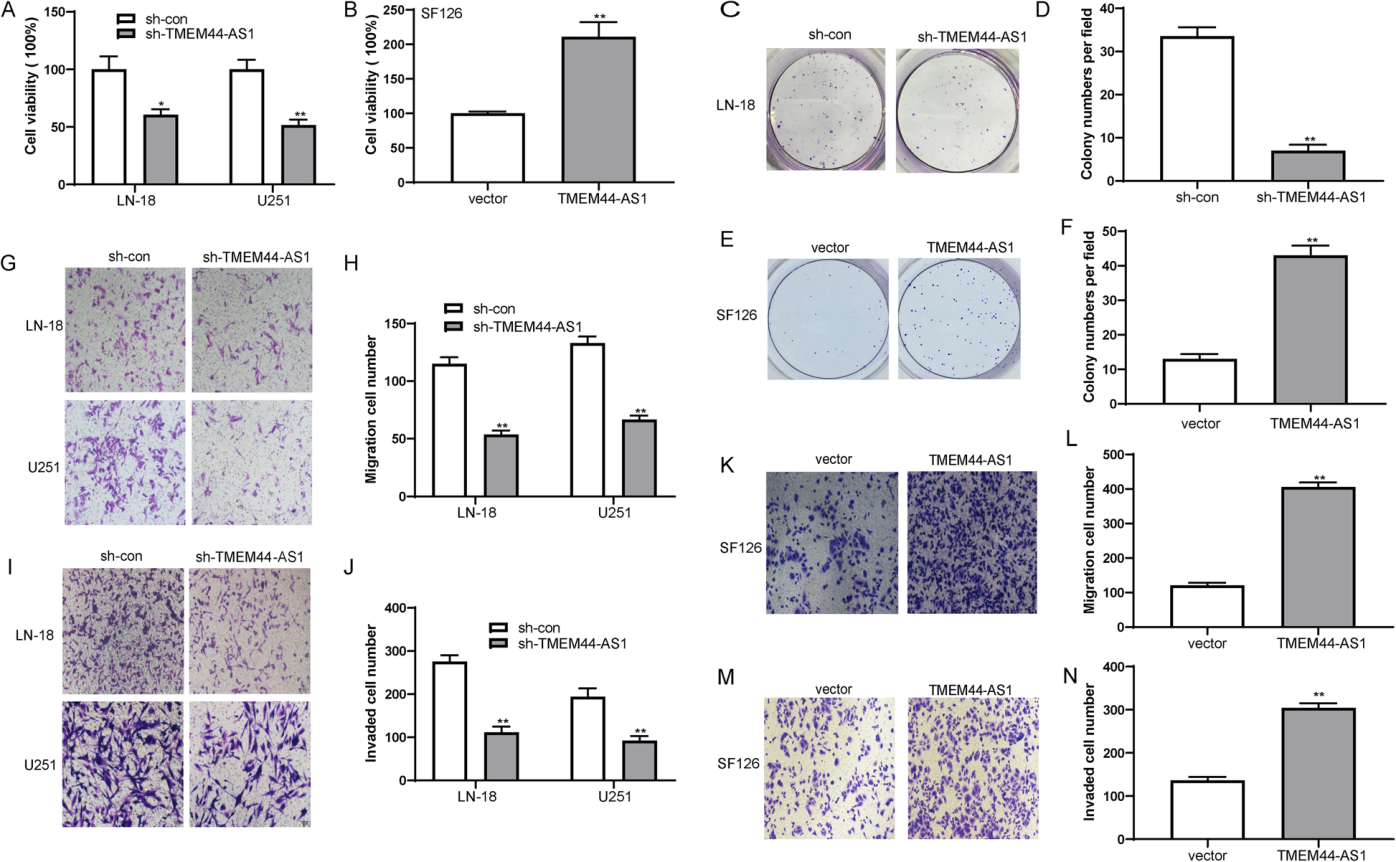
TMEM44-AS1 promotes glioma cell proliferation, colony formation, migration and invasion. (**A**) Cell viability of LN-18 and U251 cells transfected with sh-TMEM44-AS1 or sh-con was evaluated with CCK8 assay, respectively. *P < 0.05, **P < 0.01. (**B**) CCK-8 assay of SF126 cells transfected with TMEM44-AS1 plasmid or vector. **P < 0.01. (**C**-**D**) Colony formation assay of LN-18 cells transfected with sh-TMEM44-AS1 or sh-con. **P < 0.01. (**E**-**F**) Colony formation assay of SF126 cells transfected with TMEM44-AS1 or empty vector. **P < 0.01. (**G**-**J**) Representative images of transwell migration and invasion assays performed in LN-18 and U251 transfected sh-TMEM44-AS1 or sh-con cells. **P < 0.01. (**K**-**N**) Representative images of transwell migration and invasion assays in SF126 cells after transfection of sh-TMEM44-AS1 or sh-con. **P < 0.01. Data are presented as mean ± SD. The independent biological experiments were repeated at least three times

To evaluate the biofunctional role of TMEM44-AS1 in glioma cells, we interfered with endogenous TMEM44-AS1 expression by specific siRNA or lentivirus-expressing shRNA specific to TMEM44-AS1, or TMEM44-AS1-overexpressing plasmid, and then we confirmed the transfection efficiency by RT-qPCR analysis (FigS.1A-C). Consequently, cell viability was distinctly inhibited in LN-18 and U251 glioma cells transfected with sh-TMEM44-AS1 compared to the sh-con group (Fig.2A). In contrast, TMEM44-AS1 overexpression enhanced SF126 cell viability (Fig.2B). Additionally, TMEM44-AS1 knockdown resulted in a significant reduction in clone number and size compared with the sh-con group in LN-18 glioma cells (Fig.2C-D), whereas overexpression of TMEM44-AS1 increased clone numbers in SF126 glioma cells (Fig.2E-F). Meanwhile, TMEM44-AS1 knockdown significantly suppressed the cell transmigration compared with the control cells (Fig.2G-H). Knockdown of TMEM44-AS1 also markedly inhibited the invasive capabilities of glioma cells (Fig.2I-J). However, overexpression of TMEM44-AS1 could significantly enhance the migration and invasion of SF126 glioma cells (Fig.2K-N). These results suggested that TMEM44-AS1 functions as an oncogenic lncRNA in glioma.

### TMEM44-AS1 knockdown suppresses tumorigenicity in vivo

**Fig. 3:**
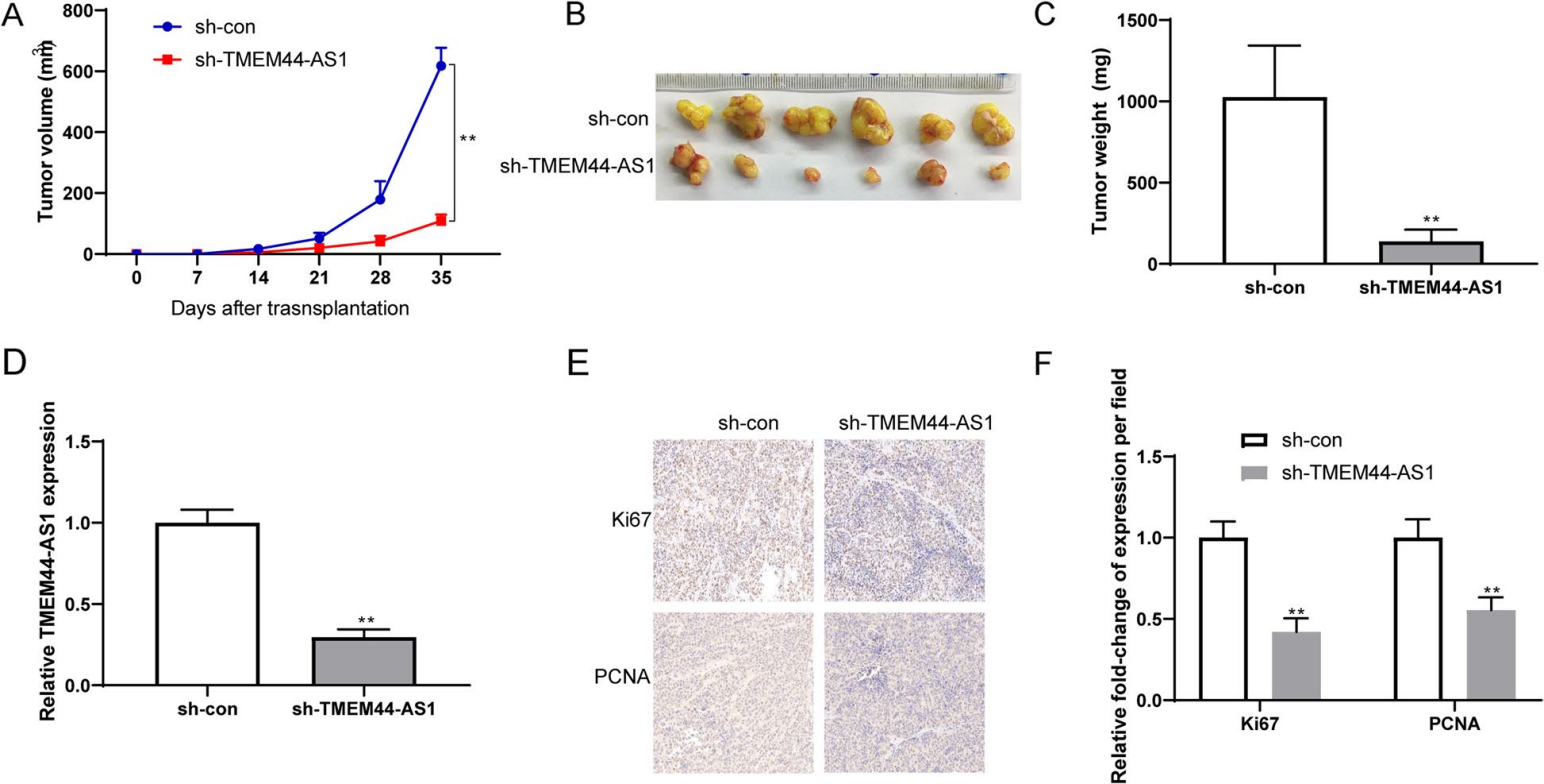
TMEM44-AS1 promotes glioma cell growth in vivo. (**A**-**C**) Glioma cells stably transfected with either sh-TMEM44-AS1 or sh-con were inoculated into the flanks of nude mice to construct a transplanted tumor model. Representative images of tumor volume growth curve and tumor weight were shown. (**D**) RT-qPCR was conducted to detect the expression levels of TMEM44-AS1 in tumor samples. **P < 0.01. (**E**) Ki67 and PCNA immunohistochemical (IHC) staining of tumor tissues of mice injected with sh-con or sh-TMEM44-AS1 cells. (**F**) The Ki67 and PCNA positive cells were qualified. **p < 0.01 vs. sh-con

To confirm that the role of TMEM44-AS1 in glioma cell growth in vivo, sh-TMEM44-AS1/sh-con transfected glioma cells were inoculated into nude mice. Compared with the sh-con group, the mice injected with sh-TMEM44-AS1 transfected glioma cells exhibited significantly smaller relative tumor volumes (Fig.3A-B). And knockdown of TMEM44-AS1 tumor weight is lower than those in the sh-con group (Fig.3C). These findings indicate that TMEM44-AS1 knockdown suppresses glioma cell growth in vivo. We confirmed that TMEM44-AS1 knockdown inhibited the TMEM44-AS1 expression in xenografted tumor tissues (Fig.3D). Glioma cell proliferation indexes were assessed by stained for proliferating cell nuclear antigen (PCNA) and Ki-67, two indicators for cell proliferative ability, in xenograft tissues. As shown in Fig.3E-F, TMEM44-AS1 knockdown had fewer PCNA and Ki-67 proliferative cells. These findings indicated that TMEM44-AS1 knockdown in vivo functions similarly to that in vitro.

### TMEM44-AS1 regulates Myc and EGR1/IL-6 in glioma

**Fig. 4:**
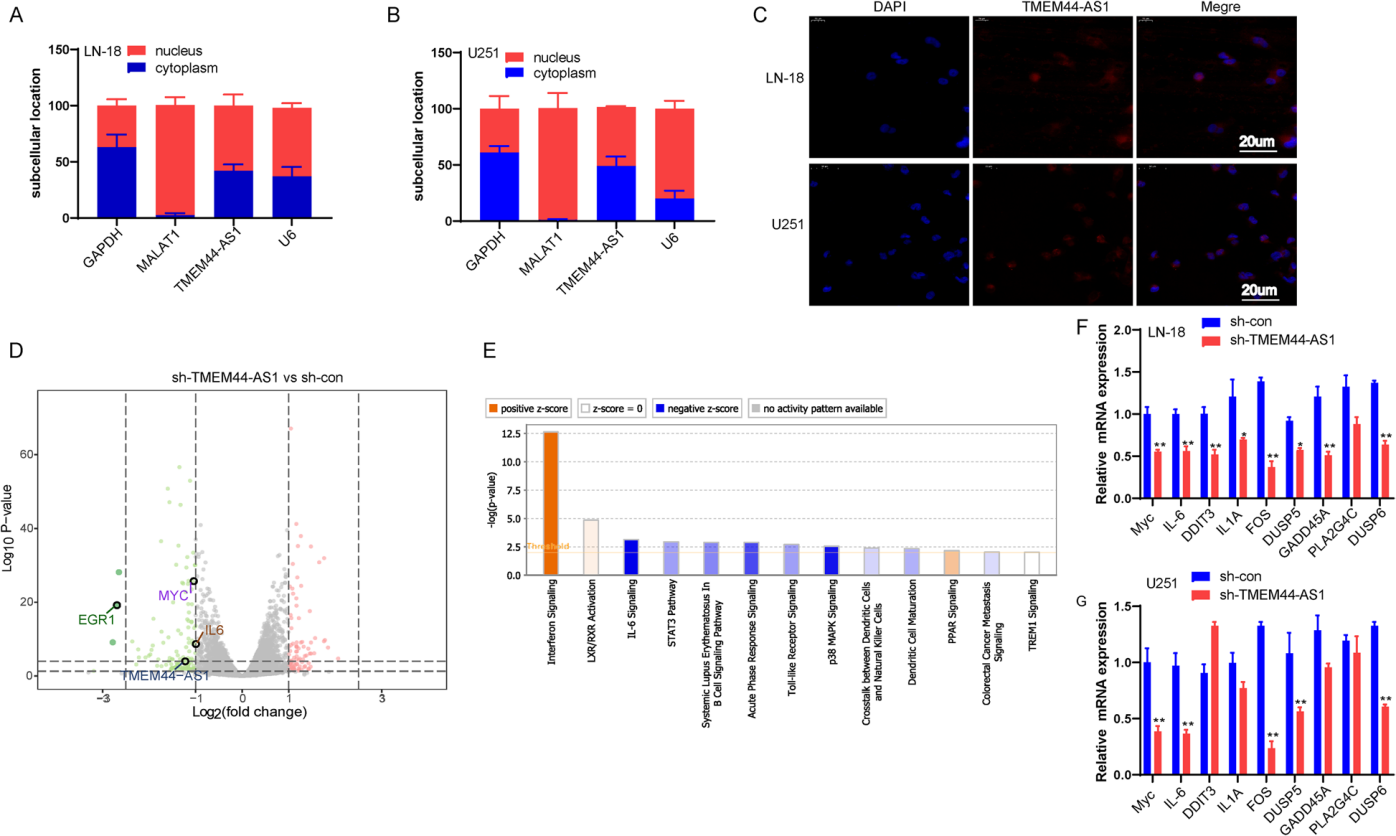
TMEM44-AS1 represses Myc translocation. (**A**-**B**) Subcellular fractionation analysis was performed in LN-18 and U251 cells. Cytoplasmic fraction (blue), and nuclear fraction (red) and probed for TMEM44-AS1. GAPDH was used as a cytoplasmic marker, U6 as a nuclear marker, and MALAT1 as the positive control. (**C**) RNA FISH assays for TMEM44-AS1. Nuclei were stained with DAPI. Scale bar = 20 μm. (**D**) Volcan representation of genes differentially expressed in LN-18 knockdown TMEM44-AS1 vs. LN-18 sh-con cells (green, downregulation; red, upregulation). (**E**) Ingenuity pathway analysis revealed that genes differentially expressed upon TMEM44-AS1 knockdown are markedly related to several cancer-associated signaling pathways. (**F**-**G**) Expression of p38MAPK and IL-6 signaling pathway associated with genes in LN-18 and U251 cells transfected with sh-TMEM44-AS1 or sh-con. *P < 0.05, **P < 0.01

Analysis of the subcellular distribution of TMEM44-AS1 by RT-qPCR analysis indicates that TMEM44-AS1 presence in the cytoplasm and nucleus of LN-18 and U251 glioma cells (Fig.4A-B). FISH confirmed that TMEM44-AS1 co-localized in the cytoplasm and nucleus of LN-18 and U251 glioma cells (Fig.4C). Since TMEM44-AS1 is slightly more prominent in the nucleus, we next focused on its potential nuclear functions in glioma cells. In a further attempt to dissect potential downstream pathways of TMEM44-AS1, we applied RNA-seq to identify genes whose expression changed after TMEM44-AS1 knockdown in LN-18 cells; 379 genes (251 downregulated and 128 upregulated) changed expression by > 2-fold in sh-TMEM44-AS1 versus sh-con-transfected cells (Fig.4D). IPA and KEGG pathway analysis revealed that the major pathways altered were the p38MAPK signaling pathway, IL-6 signaling pathway, and interferon signaling pathway (Fig.4E, Fig.2A). Among these signaling pathways, p38MAPK and IL-6 signaling, widely involved in various functions of cancer cells [19, 20], was selected further. We confirmed that p38MAPK and IL-6 signaling pathway-associated genes were inhibited in TMEM44-AS1 knockdown LN-18 and U251 glioma cells (Fig.4F-G).

**Fig. 5:**
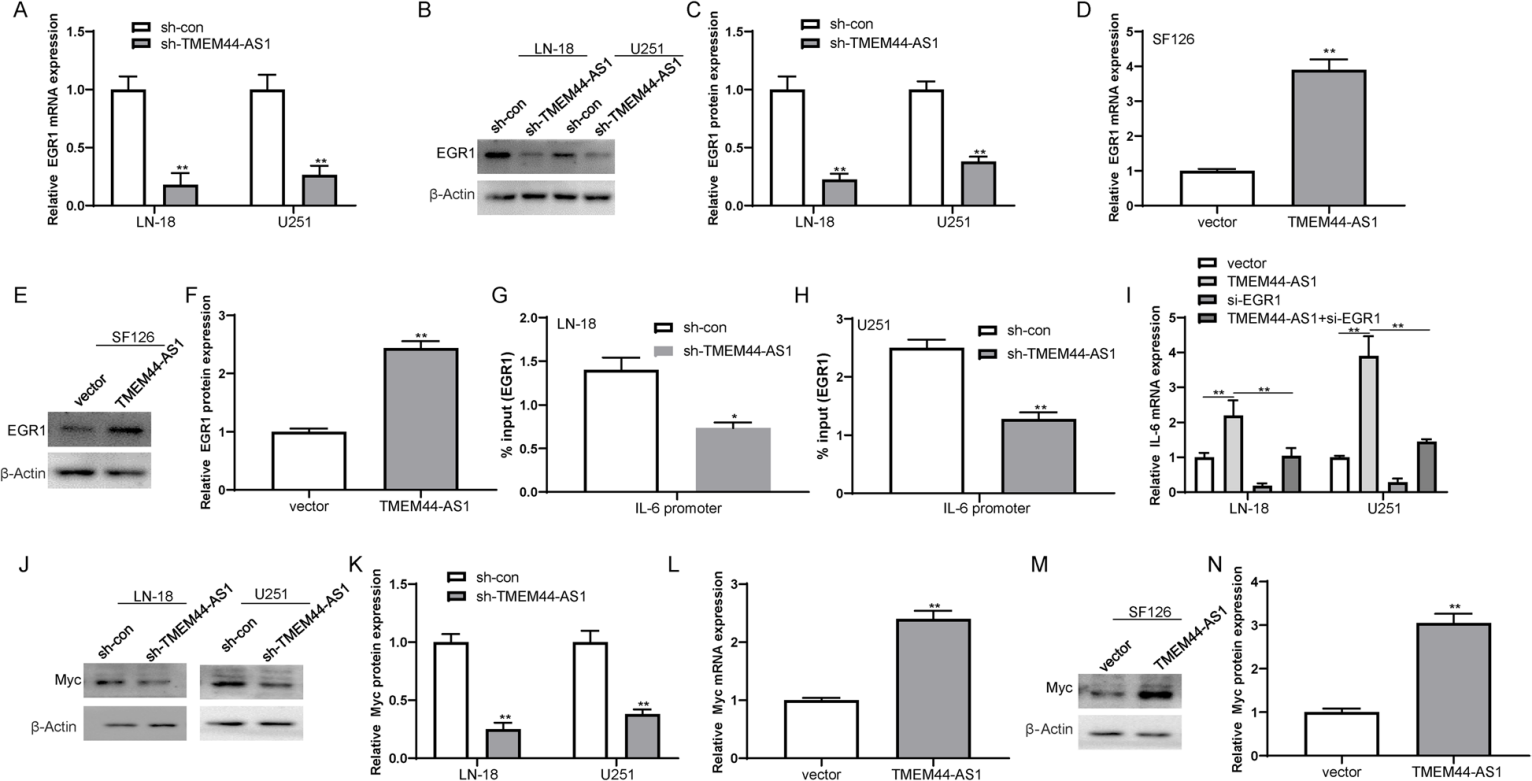
TMEM44-AS1 regulated EGR1/IL-6 and Myc signaling in glioma cells. (**A**-**C**) The expression of EGR1 upon knockdown of TMEM44-AS1 in LN-18 and U251 cells. **P < 0.01. (**D**-**F**) Expression of EGR1 in SF126 cells was examined transfected with vector or TMEM44-AS1 plasmid. **P < 0.01.(**G**-**H**) ChIP analyses of TMEM44-AS1 knockdown LN-18 and U251 cells were conducted on indicated IL-6 primer using the EGR1 antibodies. Enrichment is determined relative to input controls. *P < 0.05, **P < 0.01 vs. sh-con. (**I**) IL-6 expression in SF126 cells co-transfected with TMEM44-AS1 plasmid and si-EGR1. **p < 0.01 vs. vector; **P < 0.01 vs. TMEM44-AS1. (**J**-**K**) Expression of Myc protein in LN-18 and U251 cells transfected with sh-TMEM44-AS1 or sh-con. **P < 0.01. (**L**-**N**) Expression of Myc in SF126 cells transfected with vector or TMEM44-AS1 expression plasmid. **P < 0.01. Data are from three biological replicates represented as mean ± SD

In addition, overexpression of TMEM44-AS1 increased the IL-6 expression in SF126 glioma cells (FigS.2B). The previous study has shown that the direct binding of EGR1 to the promoter regions of genes encoding various inflammatory mediators such as IL-6 [21]. Similar to IL-6, knockdown of TMEM44-AS1 reduced the expression of EGR1 in LN-18 and U251 cells, whereas TMEM44-AS1 overexpression increased EGR1 expression in SF126 glioma cells (Fig.5A-F). Therefore, we set to investigate whether TMEM44-AS1 causes the downregulation of IL-6 by EGR1. As expected, we found that knockdown of TMEM44-AS1 reduced the recruitment of EGR1 to the promoter of IL-6 in LN-18 and U251 glioma cells (Fig.5G-H). Moreover, EGR1 knockdown partially relieved the increased expression of IL-6 induced by overexpression of TMEM44-AS1 (Fig.5I). Additionally, Myc protein expression was generally reduced in the TMEM44-AS1 knockdown LN-18 and U251 glioma cells (Fig.5J-K), whereas increased in TMEM44-AS1-overexpression SF126 glioma cells (Fig.5L-N). Taken together, the above results suggest that both Myc and EGR1/IL-6 may be important mediators of the functions of TMEM44-AS1 in glioma.

### Myc transcription activates TMEM44-AS1 in glioma

**Fig. 6:**
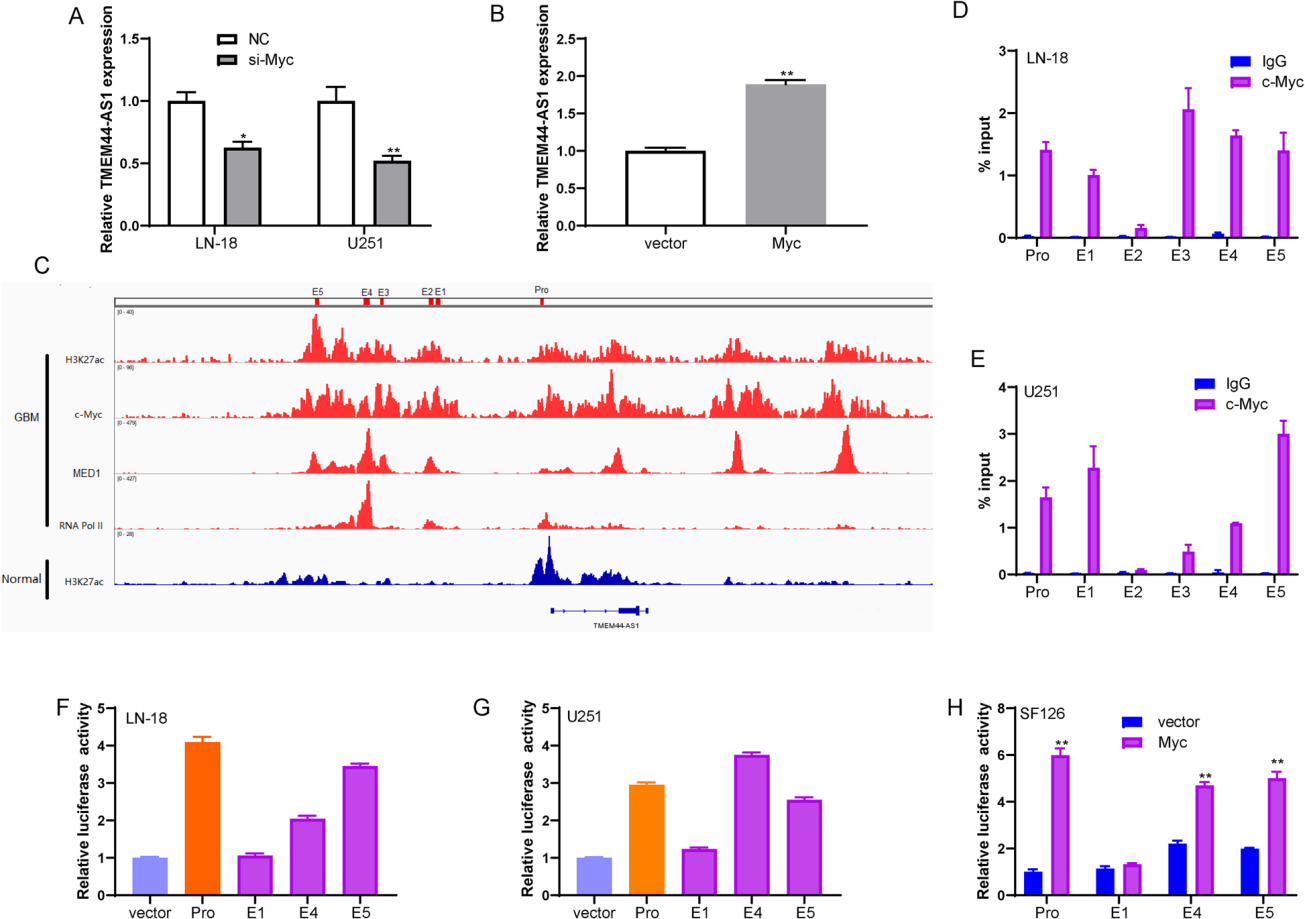
Myc bind to TMEM44-AS1 super-enhancer and promoter region, and promoted its transcription. (**A**) RT-qPCR assays measuring the expression of TMEM44-AS1 upon knockdown of Myc in LN-18 and U251 cells. *P < 0.05, **P < 0.01. (**B**) Expression of TMEM44-AS1 in SF126 cells was examined transfected with vector or Myc plasmid. **P < 0.01. (**C**) ChIP-seq profiles of H3K27ac, Myc, MED1, and RNA pol II in GBM cells and H3K27ac in normal brain tissues. (**D**-**E**) Myc ChIP followed by RT-qPCR analyzed the occupation of Myc on the super-enhancer and promoter of TMEM44-AS1. The co-immunoprecipitated DNA was amplified by PCR using indicated primer. (**F**-**G**) Enhancer and promoter activity measured by luciferase reporter assays in LN-18 and U251 cells. Three constituent enhancers (E1, E4 and E5) within the SE were separately cloned into luciferase reporter vector pGL3-enhancer and the promoter was cloned into luciferase reporter vector pGL3-promoter. (**H**) Luciferase assay analysis of SF126 cells transfected with TMEM44-AS1 promoter, or constituent enhancers (E1, E4 and E5) constructs together with Myc or vector

Interestingly, we found that TMEM44-AS1 expression was decreased by Myc knockdown in LN-18 and U251 glioma cells (Fig.6A, FigS.2C), and overexpression of Myc increased TMEM44-AS1 expression in SF126 glioma cells (Fig.6B, FigS.2D). Based on the above results, TMEM44-AS1/Myc may form the positive feedback loop in glioma. Myc gene functions as a transcriptional factor [21]. To explore how Myc affected TMEM44-AS1 expression at the transcriptional level, H3K27ac ChIP-seq data generated in glioma cells identified a super-enhancer (SE) 12 kb upstream of TMEM44-AS1 (Fig.6C). Notably, Myc is bound to both the identified promoter and super-enhancer of TMEM44-AS1 and co-localized with H3K27ac and Pol2 II in the binding region (Fig.6C). Furthermore, ChIP assay results revealed that Myc could directly bind to the TMEM44-AS1 promoter and super-enhancer in LN-18 and U251 glioma cells (Fig.6D-E). Individual constituent enhancers (E1, E4, and E5) of the TMEM44-AS1-SE and the TMEM44-AS1-pro were cloned into enhancer-reporter vectors and promoter-reporter vectors, respectively, and we then measured their luciferase activity. Promoter, E4, and E5 were active in LN-18 and U251 cells but not E1 (Fig.6F-G). As indicated in Fig.6H, luciferase reporter assays showed an increasing luciferase activity of TMEM44-AS1 promoter and enhancer in Myc-overexpression cells compared with the vector group. These findings further prove that TMEM44-AS1 promotes Myc transcription by forming a positive feedback loop with Myc in glioma.

### TMEM44-AS1 regulates Myc and EGR1 dependent on SerpinB3

**Fig. 7:**
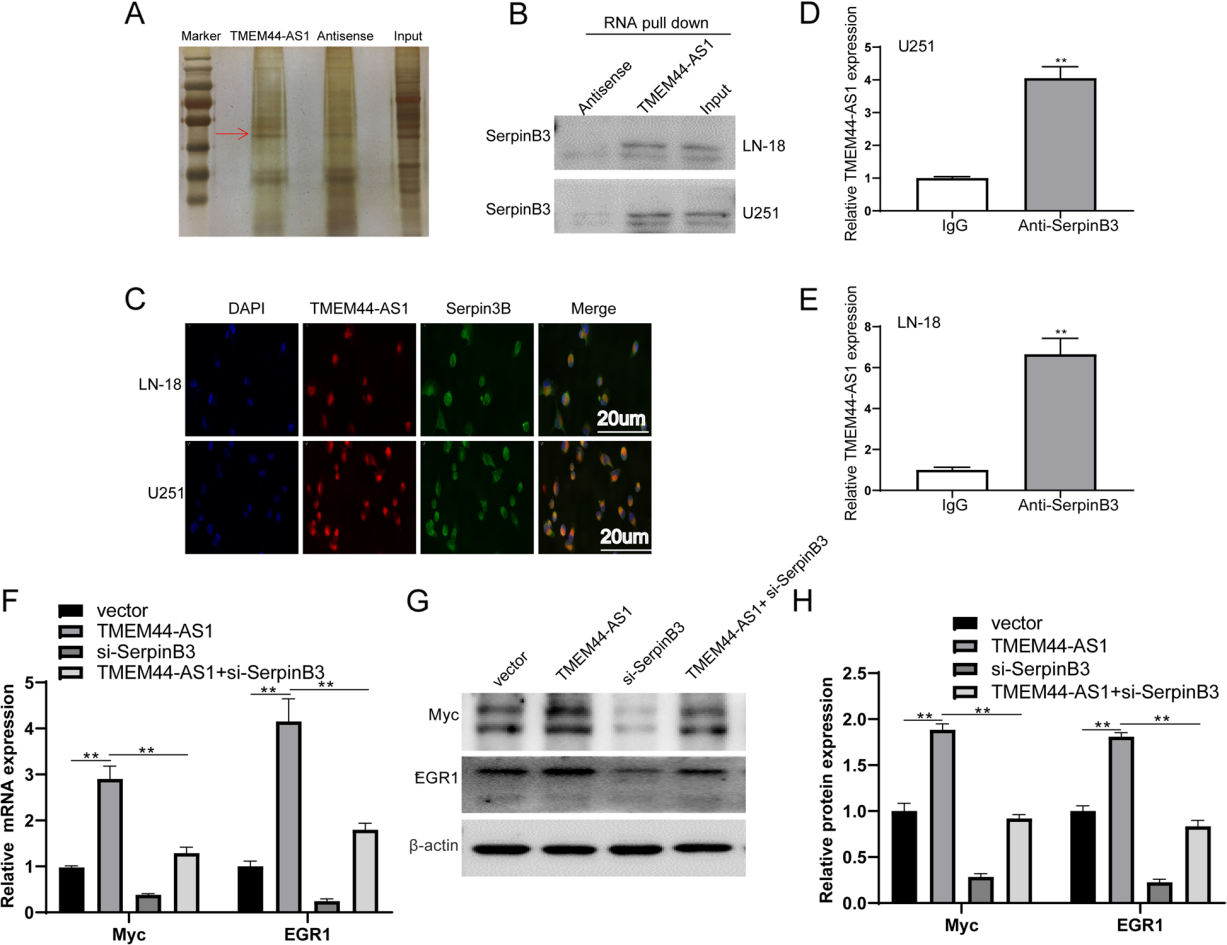
The interaction of TMEM44-AS1 and SerpinB3 activates Myc. (**A**) TMEM44-AS1 pulldown assay was performed and the associated proteins were separated with SDS-PAGE and silver staining. (**B**) SerpinB3 in TMEM44-AS1 RNA pulldown were analyzed by western blot. (**C**) Co-localization of TMEM44-AS1 and SerpinB3 in glioma cells. TMEM44-AS1 probes (red) and SerpinB3 antibody (green) were used for staining. Scale bars, 20 μm. (**D**-**E**) RNA immunoprecipitation (RIP) using the SerpinB3 antibody followed by RT-qPCR for TMEM44-AS1. (**F**-**H**) Expression of Myc and EGR1 in LN-18 and U251 cells co-transfected with TMEM44-AS1 plasmid and si-SerpinB3. **p < 0.01 vs. vector; **P < 0.01 vs. TMEM44-AS1

To further explore how TMEM44-AS1 enhances Myc and EGR1 expression, RNA-Pull down assay combined with mass spectrometry analysis was performed. These results showed that Serpin Family B Member 3 (SerpinB3) was co-precipitated with in vitro synthesized TMEM44-AS1 but not with antisense TMEM44-AS1 (Fig.7A-B). Additionally, an immunofluorescence double-labeling experiments assay was performed to identify the subcellular location of TMEM44-AS1 and SerpinB3 in glioma cells. To a large extent, TMEM44-AS1 and SerpinB3 were mainly colocalized in the cytoplasm of LN-18 and U251 glioma cells (Fig.7C). Finally, we performed RNA immunoprecipitation (RIP) assays using specific SerpinB3 antibodies in glioma cells; SerpinB3 was confirmed to interact with TMEM44-AS1 (Fig.7D-E). TMEM44-AS1 knockdown exhibited no significant effect on SerpinB3 expression (FigS.3A), indicating SerpinB3 may function as a molecular chaperon for the binding between TMEM44-AS1 and downstream targets. To further verify that TMEM44-AS1 could contribute to downstream targets Myc and EGR1 expression through SerpinB3, we co-transfect TMEM44-AS1 and si-SerpinB3, and Myc and EGR1 expression was evaluated. In accordance, ectopic TMEM44-AS1 increased Myc and EGR1 in both mRNA and protein levels, but not in SerpinB3-knockdown SF26 glioma cells (Fig.7F-H). Furthermore, knockdown of SerpinB3 in LN-18 and U251 glioma cells reduced Myc and EGR1 expression (FigS.3B-D). Collectively, these findings indicated that the effect of TMEM44-AS1 on downstream targets Myc and EGR1 may be dependent on SerpinB3.

### Myc interacts with MED1 to regulate the super-enhancer of TMEM44-AS1

**Fig. 8:**
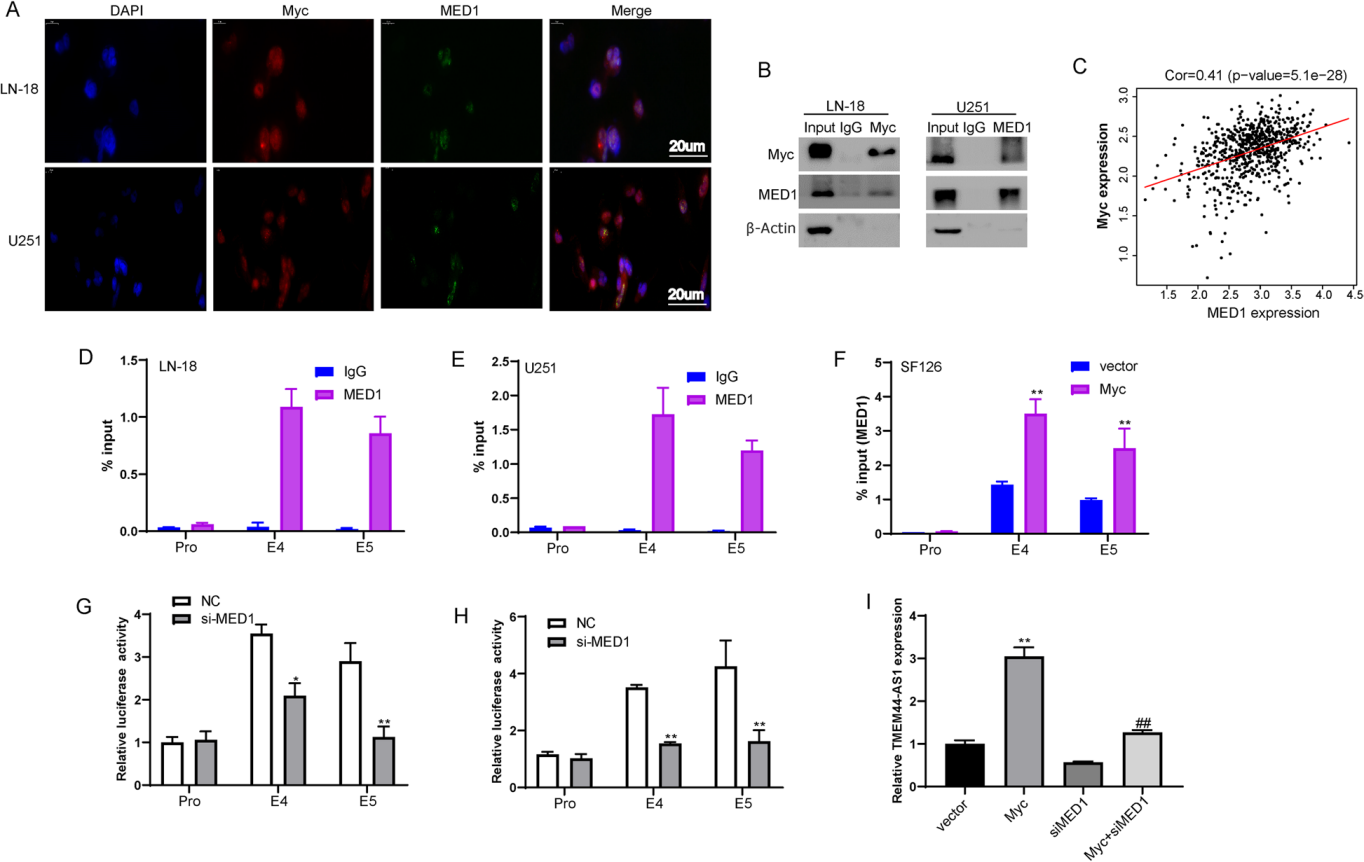
Myc affects MED1 occupancy at the TMEM44-AS1 super-enhancer in glioma cells. (**A**) Immunofluorescence staining to detect the co-localization Myc (red), and MED1 (green), and DAPI (blue) in LN-18 and U251 cells. (**B**) Co-IP assays were performed using LN-18 and U251 cells lysate with Myc or MED1 antibodies and detected by western blot with indicated antibodies. (**C**) Correlation between Myc and MED1 expression in the TCGA glioma dataset. (**D**-**E**) MED1 ChIP followed by qPCR analyzed the occupation of MED1 on the super-enhancer and promoter of TMEM44-AS1. The co-immunoprecipitated DNA was amplified by PCR using indicated primer. (**F**) The enrichment of MED1 on the enhancers of TMEM44-AS1 was analyzed with ChIP assays using the MED1 antibody after SF126 cells were transfected with vector or Myc plasmid. **P < 0.01 vs. vector. The independent biological experiments were repeated at least three times. (**G**-**H**) The luciferase reporter assays were used to detect the TMEM44-AS1 promoter, or constituent enhancers (E4 and E5) in LN-18 and U251 cells transfected with si-MED1 or NC. *P < 0.05, **P < 0.01 vs. NC. The independent biological experiments were repeated at least three times. (**I**) TMEM44-AS1 expression in LN-18 and U251 cells co-transfected with Myc plasmid and si-MED1. ^**^p < 0.01 vs. vector; ^##^P < 0.01 vs. Myc

The mediator complex subunit 1 (MED1) integrates a multiprotein complex that is required for gene transcription by the RNA polymerase II transcriptional.

machinery [22]. MED1 has been described as a transcriptional coactivator specifically enriched in a class of regulatory regions called super-enhancers [23]. Because MED1 and Myc are both enriched in super-enhancers of TMEM44-AS1 (Fig. 5C), we sought to determine whether the interaction of Myc and MED1 in regulating the TMEM44-AS1. Firstly, we examined the subcellular location of Myc and MED1 using immunofluorescence staining. Immunofluorescence imaging revealed that Myc mainly colocalized with MED1 in the nucleus of LN-18 and U251 glioma cells (Fig. 8A). Furthermore, endogenous Myc coimmunoprecipitated with endogenous MED1 in LN-18 and U251 cells (Fig.8B). The interaction between MED1 and Myc was further demonstrated by reverse endogenous coimmunoprecipitation of MED1 with Myc (Fig.8B), verifying the interaction between MED1 and Myc in vitro. Additionally, both MED1 and Myc were markedly upregulated in human glioma, associated with advanced histological grade in glioma patients (FigS.4A-D). Most importantly, Myc was also significantly correlated with MED1 expression in glioma (Fig.8C). These results prompted us to explore whether MED1 is associated with super-enhancer of TMEM44-AS1 mediated by Myc; we conducted ChIP analysis. MED1 could directly bind to the TMEM44-AS1 super-enhancer in LN-18 and U251 glioma cells, but not promoter (Fig.8D-E). Moreover, we found that Myc increased the binding of MED1 across the TMEM44-AS1 super-enhancer in SF126 glioma cells (Fig.8F). As shown in Fig.8G-H, FigS.5, decreasing luciferase activity of TMEM44-AS1 super-enhancer, but not the promoter, in MED1 knockdown LN-18 and U251 cells compared with NC cells. These findings indicate that Myc is associated with MED1 occupancy at the super-enhancer of TMEM44-AS1. To further verify whether MED1 mediated epigenetic activation of TMEM44-AS1 induced Myc, we observed TMEM44-AS1 expression after co-transfection of si-MED1 and Myc to SF126 glioma cells. As shown in Fig.8I, MED1 knockdown partially reduced TMEM44-AS1 expression induced by Myc. Together, these data indicated that MED1 might be involved in the function of Myc as an epigenetic activator of TMEM44-AS1 in glioma cells.

### Small-molecule Myc inhibitors alleviated TMEM44-AS1-promoted the growth of glioma cells

**Fig. 9:**
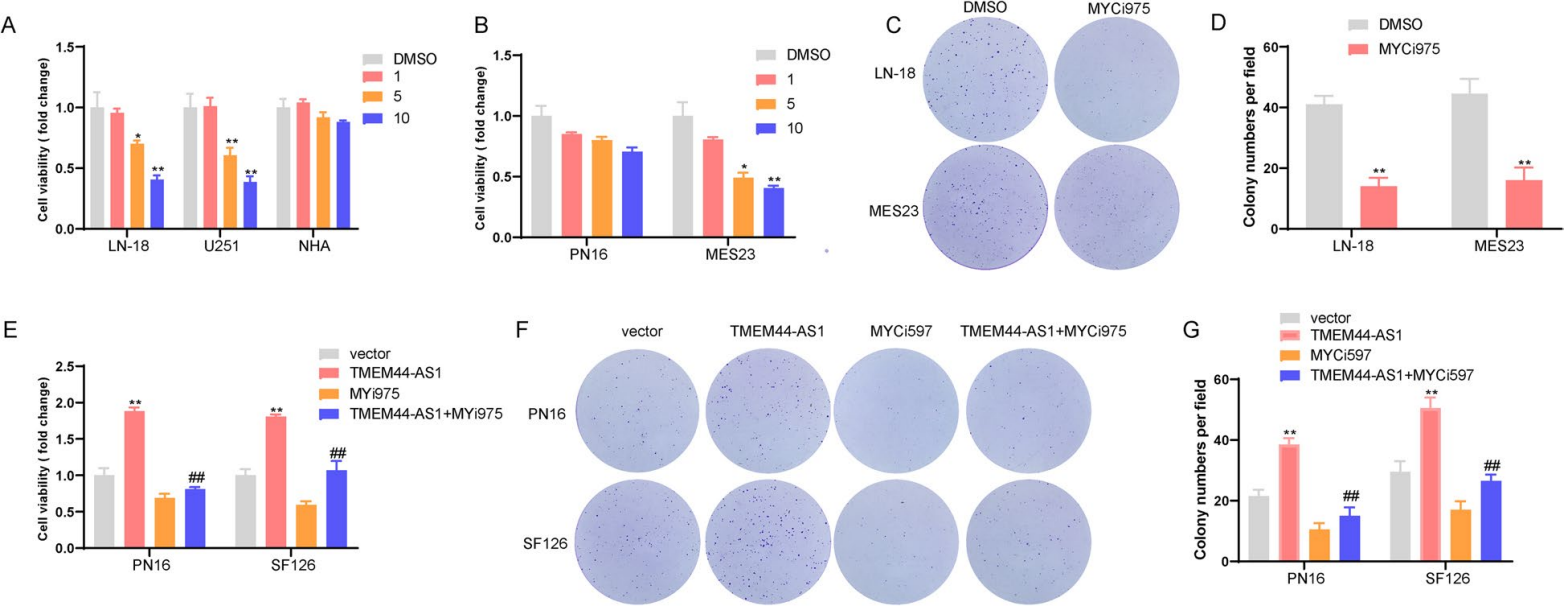
Myci975 reversed TMEM44-AS1-promoted the growth of glioma cells .(**A**) CCK-8 assay of LN-18, U251, and NHA cells treated with DMSO or Myci975 at an indicated concentration. *P < 0.05, **P < 0.01 vs. DMSO. (**B**) CCK-8 assay of PN16 and MES23 treated with DMSO or Myci975 at an indicated concentration. *P < 0.05, **P < 0.01 vs. DMSO. (**C**-**D**) Colony formation assay of LN-18 and MES23 cells treated with DMSO or Myci975 at an indicated concentration. **P < 0.01 vs. DMSO. (**E**) CCK-8 assay of PN16 and SF126 cells transfected with TMEM44-AS1 plasmid following Myci975 treatment. **P < 0.01 vs. vector, ^##^P < 0.01 vs. TMEM44-AS1. (**F**-**G**) Colony formation assay of PN16 and SF126 cells transfected with TMEM44-AS1 plasmid following Myci975 treatment. **P < 0.01 vs. vector, ^##^P < 0.01 vs. TMEM44-AS1

To address the translational potential of TMEM44-AS1, we prioritized Small-Molecule Myc inhibitors, Myci975, for further investigation based upon the positive feedback loop of TMEM44-AS1/ Myc in glioma. CCK-8 assays showed that treatment with Myci975 for 72 h markedly inhibited the viability of LN-18 and U251 glioma cells, but not NHA cells, compared with the control group in a dose-dependent manner (Fig.9A). In addition, glioma cell proliferation was related to the expression of TMEM44-AS1, with Myci975 showing an inhibitory effect (FigS.6A-B). We then confirmed the results in patient-derived glioblastoma cells (Fig.9B). Results of colony-forming assays revealed that colony numbers of LN-18 glioma cells and patient-derived glioblastoma cells (MES23) decreased remarkably following Myci975 treatment (Fig.9C-D). We next examined whether TMEM44-AS1 was required for the anti-proliferative effect of this Myci975 therapy by treating SF126 and PN16 glioma cells stably expressing TMEM44-AS1 or/ empty vector with Myci975. CCK-8 assays showed that Myci975 partially reversed the increase in the number of viable glioma cells caused by overexpression of TMEM44-AS1 (Fig.9E). As shown in Fig.9F-G, Myci975 reversed the increase in the colony numbers of glioma cells induced TMEM44-AS1 overexpression. These results are consistent with Myci975 acting partly by inhibiting the Myc/TMEM44-AS1 feedback loop in glioma cells.

## Discussion

Gliomas, the most common and aggressive form of the central nervous system’s primary brain tumor, are characterized by a high postoperative recurrence rate and poor prognosis [24]. Therefore, there is an urgent need to characterize the regulatory mechanisms underlying glioma progression and identify novel therapeutic targets to improve the outcomes for glioma. In the present study, we identified an uncharacterized lncRNA, TMEM44-AS1, as a novel oncogenic factor in glioma cells through promoting cell growth in vitro and in vivo, migration, and invasion. Mechanistically, TMEM44-AS1 could transcriptionally regulate Myc and EGR1/IL-6 signaling in glioma cells. In addition, Myc directly bound the super-enhancer and promoter region of TMEM44-AS1 and enhanced its activation; the interaction of Myc with MED1 then reciprocally triggers the epigenetic activation of TMEM44-AS1 transcription, thus forming a positive feedback loop between TMEM44-AS1 and Myc/MED1 in glioma.

SEs consist of large clusters of active transcription enhancers which drive the transcriptional activation of critical genes that define cell identity through their regulation of differentiation [25, 26]. A growing body of evidence indicates that SEs regulate tumor development and maintain the malignant characteristics of tumor cells [27]. Several protein-coding genes associated with super-enhancers have been identified in several types of cancer, including glioma. However, non-coding RNA, especially lncRNAs, associated with super-enhancers have not been identified in glioma. The combination of ROSE with high signals of H3K27ac as analyzed by ChIP-Seq is widely used to identify SEs [28]. Therefore, we identified a super-enhancer associated with TMEM44-AS1 by integrative analysis of ChIP-seq in GBM cells. TMEM44-AS1 exhibited strong oncogenic potential in glioma cells. Additionally, the upregulation of TMEM44-AS1 was related to the poor prognosis of glioma patients.

As potent regulatory components implicated in regulating genes from numerous aspects, lncRNAs can exert their effects by targeting signaling pathways [45]. In the present study, our genome-wide differential gene expression and IPA analysis show that TMEM44-AS1 knockdown inhibits p38MAPK and IL-6 signaling activation, whereas overexpression of TMEM44-AS1 exerts the opposite effect. Our RNA fractionation experiments show that TMEM44-AS1 is located in the cytoplasm and nuclear, suggesting that TMEM44-AS1 indirectly or directly induces IL-6 gene transcription. Hoffmann et al. reported that EGR1 binds to the promoter regions of IL-6 and then induced IL-6 expression [21]. Similar to IL-6, EGR1 expression was reduced in glioma cells transfected with TMEM44-AS1, whereas overexpression of TMEM44-AS1 increases EGR1 expression. Thus, we hypothesized that TMEM44-AS1 regulated IL-6 gene transcription in EGR1 dependent manner. ChIP assays show that EGR1 protein binding to the IL-6 gene promoter is reduced with TMEM44-AS1 knockdown. Moreover, EGR1 knockdown partially abrogates IL-6 expression induced by TMEM44-AS1. Therefore, these data suggest that TMEM44-AS1 indirectly induces IL-6 gene transcription through increasing protein expression of EGR1, which directly binds to the IL-6 gene promoter and enhances IL-6 gene transcription. And interestingly, we found that TMEM44-AS1 is coincidently a mediator for p38MAPK signaling in glioma cells. TMEM44-AS1 knockdown significantly inhibits p38MAPK signaling in glioma cells. Although some lncRNAs have been reported to promote MAPK signaling in the tumor, our results found that a novel lncRNA TMEM44-AS1 regulates Myc, a critical molecular of MAPK signaling glioma cells. The crucial roles of Myc signaling in glioma cell proliferation and survival have been proved by MAPK-protein-deficient mice [29, 30]. These results indicated that TMEM44-AS1 exerts its function mainly through p38MAPK and EGR1/IL-6 signaling in glioma.

Accumulating evidence has revealed that lncRNAs upregulation caused by transcriptional activation plays an essential role in tumorigenesis [31]. Transcription factors (TFs) are well-known to be critical regulators of lncRNAs expression at the transcription level [32]. Herein, we demonstrated that Myc was the major contributor to the activation of TMEM44-AS1 transcription. Myc is regarded as an archetypical proto-oncogene, and its deregulated expression is frequently related to poor prognosis in various types of human cancers, especially in glioblastoma, suggesting a pivotal role for Myc in tumorigenesis. Myc functions as a pleiotropic transcription factor that has been shown to control the expression of a large number of genes associated with cell proliferation, growth, transformation, and metabolism [33]. Furthermore, mounting evidence supports a critical role in the effect of Myc activation on cancer-cell transcriptomes [34]. In accordance, we confirmed that Myc is bound to the shared promoter and super-enhancer of TMEM44-AS1 to activate their transcription and expression. To our knowledge, it is the first report linking the Myc directly to super-enhancer activation of TMEM44-AS1 via changes in epigenetic modifications. Our study helps explain why the Myc oncoprotein can result in transcriptional activation of the oncogene. Additional studies need to be performed to clarify the mechanisms involved and how these unique layers of the epigenome/genome/transcriptome (lncRNAs, enhancers, and SEs) co-operate during Myc mediated gene expression [35].

Previous studies have demonstrated that lncRNA PCGM1, GHET1, CCAT1-L, and PVT1 regulate Myc transcription or RNA and protein stability in the prostate, gastric, and colorectal cancers [36–39]. However, little is known about how lncRNAs regulate the transcriptional activity of Myc. Depending on their subcellular distribution, lncRNAs exert their functions by different mechanisms [40]. Nuclear lncRNAs can interact with chromatin and regulate the expression of the gene at the transcriptional level, while cytoplasmic lncRNAs can bind to large biomolecules, such as RNAs or proteins [41]. TMEM44-AS1 was located in both the cytoplasm and nucleus, indicating that TMEM44-AS1 could mediate the expression of target genes in different ways. In the present study, we showed that TMEM44-AS1 and SerpinB3 mainly interact in the cytoplasm of glioma cells. Previous studies reported that SerpinB3 contributes to tumorigenesis by allowing Myc nuclear translocation and enhancing IL-6 signaling [42, 43]. Thus, we investigated whether TMEM44-AS1 affected Myc and EGR1/IL-6 signaling in a manner dependent on or independent of SerpinB3. Our results confirmed that TMEM44-AS1 promoted Myc and EGR1/IL-6 activation in a manner that might be dependent upon SerpinB3. Although we cannot exclude that other genetic or epigenetic factors regulate Myc, this is the only study to indicate that SerpinB3 is involved in Myc regulated by TMEM44-AS1. Briefly, TMEM44-AS1 is a lncRNA capable of the interaction of SerpinB3 and further increasing the activation of c-Myc signaling in glioma cells. However, the following several questions may require further addressed: How does TMEM44-AS1 affect SerpinB3? How does TMEM44-AS1 identify and interact with precise genomic target sites of SerpinB3? In future work, we will focus on addressing the aforementioned issues.

**Fig. 10:**
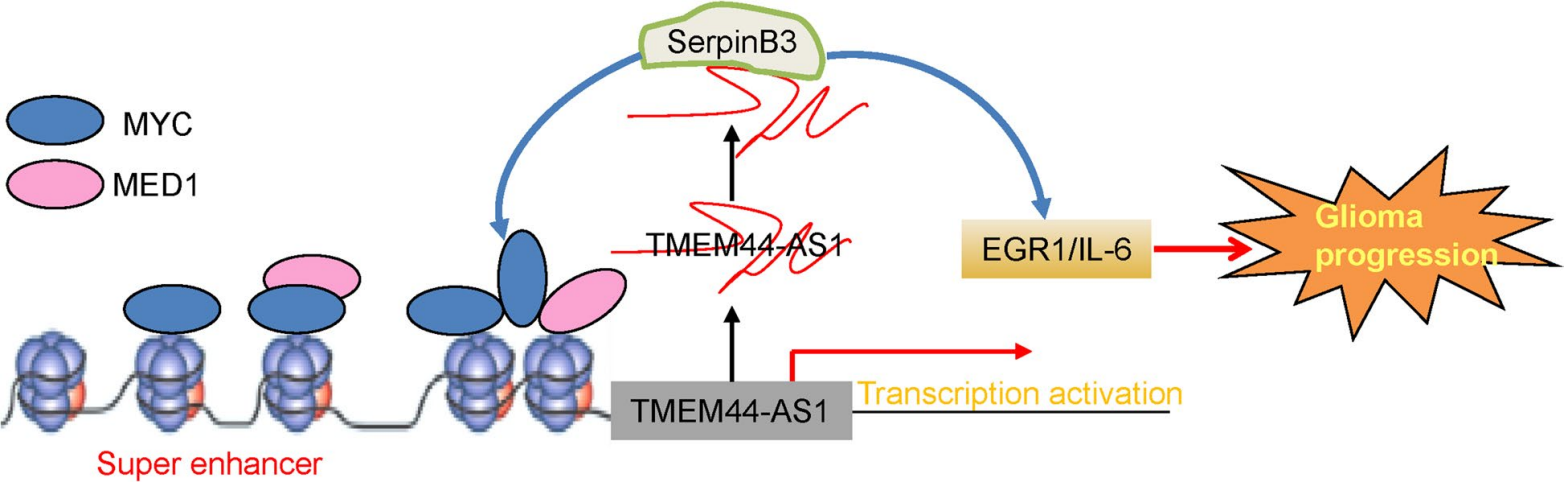
Depiction of the proposed signaling network involved in the regulation of super-enhancer associated-TMEM44-AS1 in glioma progression

Myc functions as a TF by cooperation with its cofactors [44]. Activation or suppression of Myc-regulated genes is mediated through interaction with other protein partners involved in chromatin structure regulation [45]. It is believed that these pioneer TFs orchestrate the accessible chromatin landscape by recruiting histone-modifying enzymes to reshape the epigenetic landscape and establish an oncogenic state [46]. These TFs may play a critical role in creating tissue-specific active enhancer sites [47]. To identify how Myc regulates TMEM44-AS1 transcription, we queried H3K27ac and MED1 ChIP-seq in GBM cells from public datasets. Interestingly, we found that H3K27ac and MED1 occupy in SE of TMEM44-AS1. The occupancy of MED1 was used as corroborative evidence to identify enhancer elements because enhancer-bound TFs and the mediator directly bind to the enhancer region of the target gene [23]. MED1 is one cofactor that promotes transcription initiation as the bridge between enhancers and promoters, interacting preferentially with enhancer-bound TFs localized at the enhancers and interacting transiently with the Pol II initiation complex at the promoter [48]. In addition, it has been shown that MED1 is highly enriched in the super-enhancer region in embryonic stem cells, and reduced levels of mediator subunits cause preferential loss of expression of super-enhancer-associated genes [49]. This paper found that Myc co-localized with MED1 recruited MED1 to the super-enhancer of TMEM44-AS1 and then activated TMEM44-AS1. A recent study showed that MED1 mainly binds to the enhancer and promoter regions of target genes to promote transcriptional activation of the gene [50]. However, we found that Myc activates gene transcription by interacting with MED1 at enhancers, not the promoter. Thus, our findings indicate that Myc mediates the activation of TMEM44-AS1 transcription partly by recruiting MED1 to the enhancer of the target gene, and the Myc-MED1 interaction may be critical for adequate transcription of gene (Fig.10). This study might have the following limitations. Despite the observed phenotypic effects, further research may need to investigate whether Myc’s regulatory impact on TMEM44-AS1 expression is independent of the MED1. Additional factors need to be identified the underlying mechanism of TMEM44-AS1’s ability to regulate the Myc autoregulatory loop and evaluate itself could be a component of this regulatory network. To address the translational potential of TMEM44-AS1/Myc, we identified the Myc inhibitor, Myci975, as a selective inhibitor of glioma cell growth, which further validates our finding that Myc is a downstream effector of TMEM44-AS1. Myci975 has been reported to have significant in vivo anti-tumor efficacy [51], although it has not yet been tested in glioma. Our results support further investigation into the clinical utility of Myci975 and other Myc inhibitors in glioma.

## Conclusions

In summary, our findings provide solid evidence to support the idea that epigenetic activation of TMEM44-AS1 mediated by MED1 promotes glioma progression through the Myc positive feedback loop. Understanding the precise role of TMEM44-AS1 in glioma and activation of the Myc signaling pathway will improve our knowledge about the progression of human gliomas and enable novel therapeutic strategies against glioma.

## Supplementary Information

**Additional file 1:.**

**Additional file 2:.**

**Additional file 3:.**

**Additional file 4:.**

**Additional file 5:.**

**Additional file 6:.**

**Additional file 7:.**

## Data Availability

Additional data are available as Supplementary information.

## Abbreviations

GBM: Glioblastoma
lncRNA: Long non-coding RNA
SEs: super-enhancers
SerpinB3: Serpin Family B Member 3
RIP: RNA immunoprecipitation
MED1: mediator complex subunit 1
TFs: Transcription factors
LGG: low-grade tumors
siRNA: small interfering RNA
ChIP: Chromatin immunoprecipitation
Co-IP: Co-immunoprecipitation

## References

[1] Wen PY, Kesari S (2008). Malignant gliomas in adults. N Engl J Med.

[2] Louis DN, Perry A, Reifenberger G, von Deimling A, Figarella-Branger D, Cavenee WK, Ohgaki H, Wiestler OD, Kleihues P, Ellison DW (2016). The 2016 World Health Organization classification of tumors of the central nervous system: a summary. Acta Neuropathol.

[3] F. Graus, Bruna J., Pardo J., Escudero D., Vilas D., Barcelo I., Brell M., Pascual C., Crespo J. A., Erro E., Garcia-Romero J. C., Estela J., Martino J., Garcia-Castano A., Mata E., Lema M., Gelabert M., Fuentes R., Perez P., Manzano A., Aguas J., Belenguer A., Simon A., Henriquez I., Murcia M., Vivanco R., Rojas-Marcos I., Munoz-Carmona D., Navas I., de Andres P., Mas G., Gil M., Verger E. Patterns of care and outcome for patients with glioblastoma diagnosed during 2008-2010 in Spain. Neuro Oncol.2013; 15(6):797-805. 10.1093/neuonc/not013 PMC3661097 23460319

[4] Mikheeva SA, Mikheev AM, Petit A, Beyer R, Oxford RG, Khorasani L, Maxwell JP, Glackin CA, Wakimoto H, Gonzalez-Herrero I, Sanchez-Garcia I, Silber JR, Horner PJ, Rostomily RC (2010). TWIST1 promotes invasion through mesenchymal change in human glioblastoma. Mol Cancer.

[5] Huang Z, Zhou JK, Peng Y, He W, Huang C (2020). The role of long noncoding RNAs in hepatocellular carcinoma. Mol Cancer.

[6] McCabe EM (2020). Rasmussen T.

[7] Kalna V, Yang Y, Peghaire CR, Frudd K, Hannah R, Shah AV, Osuna AL, Boyle JJ, Gottgens B, Ferrer J, Randi AM, Birdsey GM (2019). The transcription factor ERG regulates super-enhancers associated with an endothelial-specific gene expression program. Circ Res.

[8] Sanchez GJ, Richmond PA, Bunker EN, Karman SS, Azofeifa J, Garnett AT, Xu Q, Wheeler GE, Toomey CM, Zhang Q, Dowell RD, Liu X (2018). Genome-wide dose-dependent inhibition of histone deacetylases studies reveal their roles in enhancer remodeling and suppression of oncogenic super-enhancers. Nucleic Acids Res.

[9] K. Liu, Gao L., Ma X., Huang J. J., Chen J., Zeng L., Ashby C. R., Jr., Zou C., Chen Z. S. Long non-coding RNAs regulate drug resistance in cancer. Mol Cancer.2020; 19(1):54. 10.1186/s12943-020-01162-0 PMC7066752 32164712

[10] X. Agirre, Meydan C., Jiang Y., Garate L., Doane A. S., Li Z., Verma A., Paiva B., Martin-Subero J. I., Elemento O., Mason C. E., Prosper F., Melnick A. Long non-coding RNAs discriminate the stages and gene regulatory states of human humoral immune response. Nat Commun.2019; 10(1):821. 10.1038/s41467-019-08679-z PMC6379396 30778059

[11] L. Richart, Carrillo-de Santa Pau E., Rio-Machin A., de Andres M. P., Cigudosa J. C., Lobo V. J. S., Real F. X. BPTF is required for c-MYC transcriptional activity and in vivo tumorigenesis. Nat Commun.2016; 7(10153. 10.1038/ncomms10153 PMC4728380 26729287

[12] Herms JW, von Loewenich FD, Behnke J, Markakis E, Kretzschmar HA (1999). C-myc oncogene family expression in glioblastoma and survival. Surg Neurol.

[13] Fomchenko EI, Holland EC (2006). Mouse models of brain tumors and their applications in preclinical trials. Clin Cancer Res.

[14] D. Annibali, Whitfield J. R., Favuzzi E., Jauset T., Serrano E., Cuartas I., Redondo-Campos S., Folch G., Gonzalez-Junca A., Sodir N. M., Masso-Valles D., Beaulieu M. E., Swigart L. B., Mc Gee M. M., Somma M. P., Nasi S., Seoane J., Evan G. I., Soucek L. Myc inhibition is effective against glioma and reveals a role for Myc in proficient mitosis. Nat Commun.2014; 5(4632. 10.1038/ncomms5632 PMC4143920 25130259

[15] Lin CY, Loven J, Rahl PB, Paranal RM, Burge CB, Bradner JE, Lee TI, Young RA (2012). Transcriptional amplification in tumor cells with elevated c-Myc. Cell..

[16] Zeid R, Lawlor MA, Poon E, Reyes JM, Fulciniti M, Lopez MA, Scott TG, Nabet B, Erb MA, Winter GE, Jacobson Z, Polaski DR, Karlin KL, Hirsch RA, Munshi NP, Westbrook TF, Chesler L, Lin CY, Bradner JE (2018). Enhancer invasion shapes MYCN-dependent transcriptional amplification in neuroblastoma. Nat Genet.

[17] Chen X, Hu L, Yang H, Ma H, Ye K, Zhao C, Zhao Z, Dai H, Wang H, Fang Z (2019). DHHC protein family targets different subsets of glioma stem cells in specific niches. J Exp Clin Cancer Res.

[18] Weber RG, Sabel M, Reifenberger J, Sommer C, Oberstrass J, Reifenberger G, Kiessling M, Cremer T (1996). Characterization of genomic alterations associated with glioma progression by comparative genomic hybridization. Oncogene..

[19] Kim YW, Liu TJ, Koul D, Tiao N, Feroze AH, Wang J, Powis G, Yung WK (2011). Identification of novel synergistic targets for rational drug combinations with PI3 kinase inhibitors using siRNA synthetic lethality screening against GBM. Neuro-Oncology.

[20] Lamano JB, Li YD, DiDomenico JD, Choy W, Veliceasa D, Oyon DE, Fakurnejad S, Ampie L, Kesavabhotla K, Kaur R, Kaur G, Biyashev D, Unruh DJ, Horbinski CM, James CD, Parsa AT, Bloch O (2019). Glioblastoma-derived IL6 induces immunosuppressive peripheral myeloid cell PD-L1 and promotes tumor growth. Clin Cancer Res.

[21] R. Dhanasekaran, Deutzmann A., Mahauad-Fernandez W. D., Hansen A. S., Gouw A. M., Felsher D. W. The MYC oncogene - the grand orchestrator of cancer growth and immune evasion. Nat Rev Clin Oncol.2021; 10.1038/s41571-021-00549-2 PMC9083341 34508258

[22] Yoshizawa T, Nozawa RS, Jia TZ, Saio T, Mori E (2020). Biological phase separation: cell biology meets biophysics. Biophys Rev.

[23] Loven J, Hoke HA, Lin CY, Lau A, Orlando DA, Vakoc CR, Bradner JE, Lee TI, Young RA (2013). Selective inhibition of tumor oncogenes by disruption of super-enhancers. Cell..

[24] Lim M, Xia Y, Bettegowda C, Weller M (2018). Current state of immunotherapy for glioblastoma. Nat Rev Clin Oncol.

[25] E. Bell, Curry E. W., Megchelenbrink W., Jouneau L., Brochard V., Tomaz R. A., Mau K. H. T., Atlasi Y., de Souza R. A., Marks H., Stunnenberg H. G., Jouneau A., Azuara V. Dynamic CpG methylation delineates subregions within super-enhancers selectively decommissioned at the exit from naive pluripotency. Nat Commun.2020; 11(1):1112. 10.1038/s41467-020-14916-7 PMC7048827 32111830

[26] S. Kubota, Tokunaga K., Umezu T., Yokomizo-Nakano T., Sun Y., Oshima M., Tan K. T., Yang H., Kanai A., Iwanaga E., Asou N., Maeda T., Nakagata N., Iwama A., Ohyashiki K., Osato M., Sashida G. Lineage-specific RUNX2 super-enhancer activates MYC and promotes the development of blastic plasmacytoid dendritic cell neoplasm. Nat Commun.2019; 10(1):1653. 10.1038/s41467-019-09710-z PMC6458132 30971697

[27] Y. Jia, Chng W. J., Zhou J. Super-enhancers: critical roles and therapeutic targets in hematologic malignancies. J Hematol Oncol.2019; 12(1):77. 10.1186/s13045-019-0757-y PMC6636097 31311566

[28] Whyte WA, Orlando DA, Hnisz D, Abraham BJ, Lin CY, Kagey MH, Rahl PB, Lee TI, Young RA (2013). Master transcription factors and mediator establish super-enhancers at key cell identity genes. Cell..

[29] Gramling MW, Eischen CM (2012). Suppression of Ras/Mapk pathway signaling inhibits Myc-induced lymphomagenesis. Cell Death Differ.

[30] Tateishi K, Iafrate AJ, Ho Q, Curry WT, Batchelor TT, Flaherty KT, Onozato ML, Lelic N, Sundaram S, Cahill DP, Chi AS, Wakimoto H (2016). Myc-driven glycolysis is a therapeutic target in Glioblastoma. Clin Cancer Res.

[31] Wu G, Hao C, Qi X, Nie J, Zhou W, Huang J, He Q (2020). LncRNA SNHG17 aggravated prostate cancer progression through regulating its homolog SNORA71B via a positive feedback loop. Cell Death Dis.

[32] Xu J, Cao X (2019). Long noncoding RNAs in the metabolic control of inflammation and immune disorders. Cell Mol Immunol.

[33] T. M. Popay, Wang J., Adams C. M., Howard G. C., Codreanu S. G., Sherrod S. D., McLean J. A., Thomas L. R., Lorey S. L., Machida Y. J., Weissmiller A. M., Eischen C. M., Liu Q., Tansey W. P. MYC regulates ribosome biogenesis and mitochondrial gene expression programs through its interaction with host cell factor-1. Elife.2021; 10(. 10.7554/eLife.60191 PMC7793627 33416496

[34] Elkon R, Loayza-Puch F, Korkmaz G, Lopes R, van Breugel PC, Bleijerveld OB, Altelaar AF, Wolf E, Lorenzin F, Eilers M, Agami R (2015). Myc coordinates transcription and translation to enhance transformation and suppress invasiveness. EMBO Rep.

[35] S. Das, Senapati P., Chen Z., Reddy M. A., Ganguly R., Lanting L., Mandi V., Bansal A., Leung A., Zhang S., Jia Y., Wu X., Schones D. E., Natarajan R. Regulation of angiotensin II actions by enhancers and super-enhancers in vascular smooth muscle cells. Nat Commun.2017; 8(1):1467. 10.1038/s41467-017-01629-7 PMC5684340 29133788

[36] Xiang JF, Yin QF, Chen T, Zhang Y, Zhang XO, Wu Z, Zhang S, Wang HB, Ge J, Lu X, Yang L, Chen LL (2014). Human colorectal cancer-specific CCAT1-L lncRNA regulates long-range chromatin interactions at the MYC locus. Cell Res.

[37] Yang F, Xue X, Zheng L, Bi J, Zhou Y, Zhi K, Gu Y, Fang G (2014). Long non-coding RNA GHET1 promotes gastric carcinoma cell proliferation by increasing c-Myc mRNA stability. FEBS J.

[38] Hung CL, Wang LY, Yu YL, Chen HW, Srivastava S, Petrovics G, Kung HJ (2014). A long noncoding RNA connects c-Myc to tumor metabolism. Proc Natl Acad Sci U S A.

[39] Tseng YY, Moriarity BS, Gong W, Akiyama R, Tiwari A, Kawakami H, Ronning P, Reuland B, Guenther K, Beadnell TC, Essig J, Otto GM, O'Sullivan MG, Largaespada DA, Schwertfeger KL, Marahrens Y, Kawakami Y, Bagchi A (2014). PVT1 dependence in cancer with MYC copy-number increase. Nature..

[40] Zarkou V, Galaras A, Giakountis A, Hatzis P (2018). Crosstalk mechanisms between the WNT signaling pathway and long non-coding RNAs. Noncoding RNA Res.

[41] Y. Qin, Hou Y., Liu S., Zhu P., Wan X., Zhao M., Peng M., Zeng H., Li Q., Jin T., Cui X., Liu M. A Novel Long Non-Coding RNA lnc030 Maintains Breast Cancer Stem Cell Stemness by Stabilizing SQLE mRNA and Increasing Cholesterol Synthesis. Adv Sci (Weinh).2021; 8(2):2002232. 10.1002/advs.202002232 PMC7816696 33511005

[42] C. Turato, Cannito S., Simonato D., Villano G., Morello E., Terrin L., Quarta S., Biasiolo A., Ruvoletto M., Martini A., Fasolato S., Zanus G., Cillo U., Gatta A., Parola M., Pontisso P. SerpinB3 and Yap Interplay Increases Myc Oncogenic Activity. Sci Rep.2015;5:17701. 10.1038/srep17701 PMC4669520 26634820

[43] Sheshadri N, Catanzaro JM, Bott AJ, Sun Y, Ullman E, Chen EI, Pan JA, Wu S, Crawford HC, Zhang J, Zong WX (2014). SCCA1/SERPINB3 promotes oncogenesis and epithelial-mesenchymal transition via the unfolded protein response and IL6 signaling. Cancer Res.

[44] Caforio M, Sorino C, Iacovelli S, Fanciulli M, Locatelli F, Folgiero V (2018). Recent advances in searching c-Myc transcriptional cofactors during tumorigenesis. J Exp Clin Cancer Res.

[45] Wolfer A, Ramaswamy S (2011). MYC and metastasis. Cancer Res.

[46] Ke L, Zhou H, Wang C, Xiong G, Xiang Y, Ling Y, Khabir A, Tsao GS, Zeng Y, Zeng M, Busson P, Kieff E, Guo X, Zhao B (2017). Nasopharyngeal carcinoma super-enhancer-driven ETV6 correlates with prognosis. Proc Natl Acad Sci U S A.

[47] Liu Z, Legant WR, Chen BC, Li L, Grimm JB, Lavis LD, Betzig E, Tjian R (2014). 3D imaging of Sox2 enhancer clusters in embryonic stem cells. Elife.

[48] Kuras L, Borggrefe T, Kornberg RD (2003). Association of the Mediator complex with enhancers of active genes. Proc Natl Acad Sci U S A.

[49] Belorusova AY, Bourguet M, Hessmann S, Chalhoub S, Kieffer B, Cianferani S, Rochel N (2020). Molecular determinants of MED1 interaction with the DNA bound VDR-RXR heterodimer. Nucleic Acids Res.

[50] Siersbaek R, Madsen JGS, Javierre BM, Nielsen R, Bagge EK, Cairns J, Wingett SW, Traynor S, Spivakov M, Fraser P, Mandrup S (2017). Dynamic rewiring of promoter-anchored chromatin loops during adipocyte differentiation. Mol Cell.

[51] Huarte M (2015). The emerging role of lncRNAs in cancer. Nat Med.

