# Supplementary figures and images for: Super-enhancer-associated TMEM44-AS1 aggravated glioma progression by forming a positive feedback loop with Myc
Source: J Exp Clin Cancer Res. 2021 Oct 25;40:337. doi: 10.1186/s13046-021-02129-9 (PMC8543865; doi:10.1186/s13046-021-02129-9)

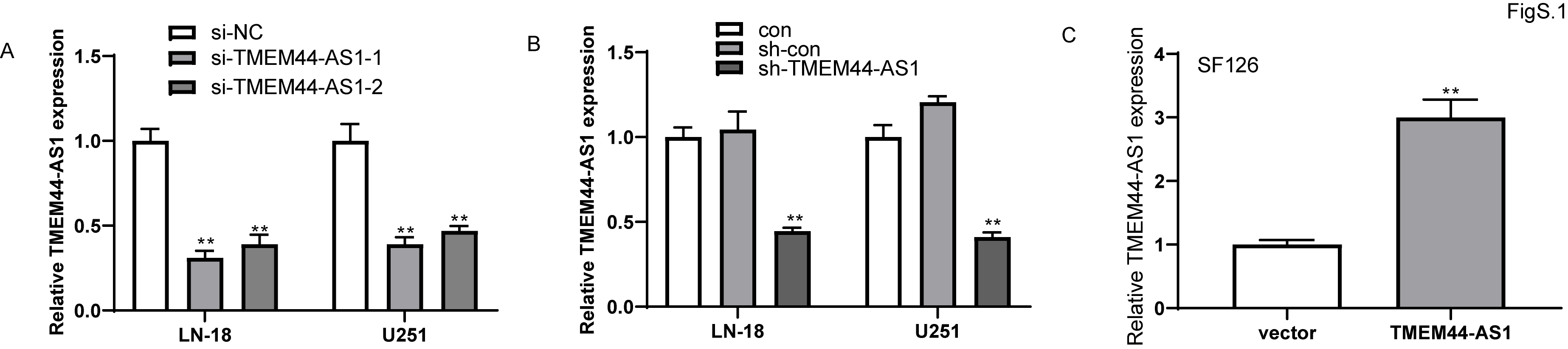

Supplement: Supplementary file 1 — Additional file 1:. [file 13046_2021_2129_MOESM1_ESM.tif]

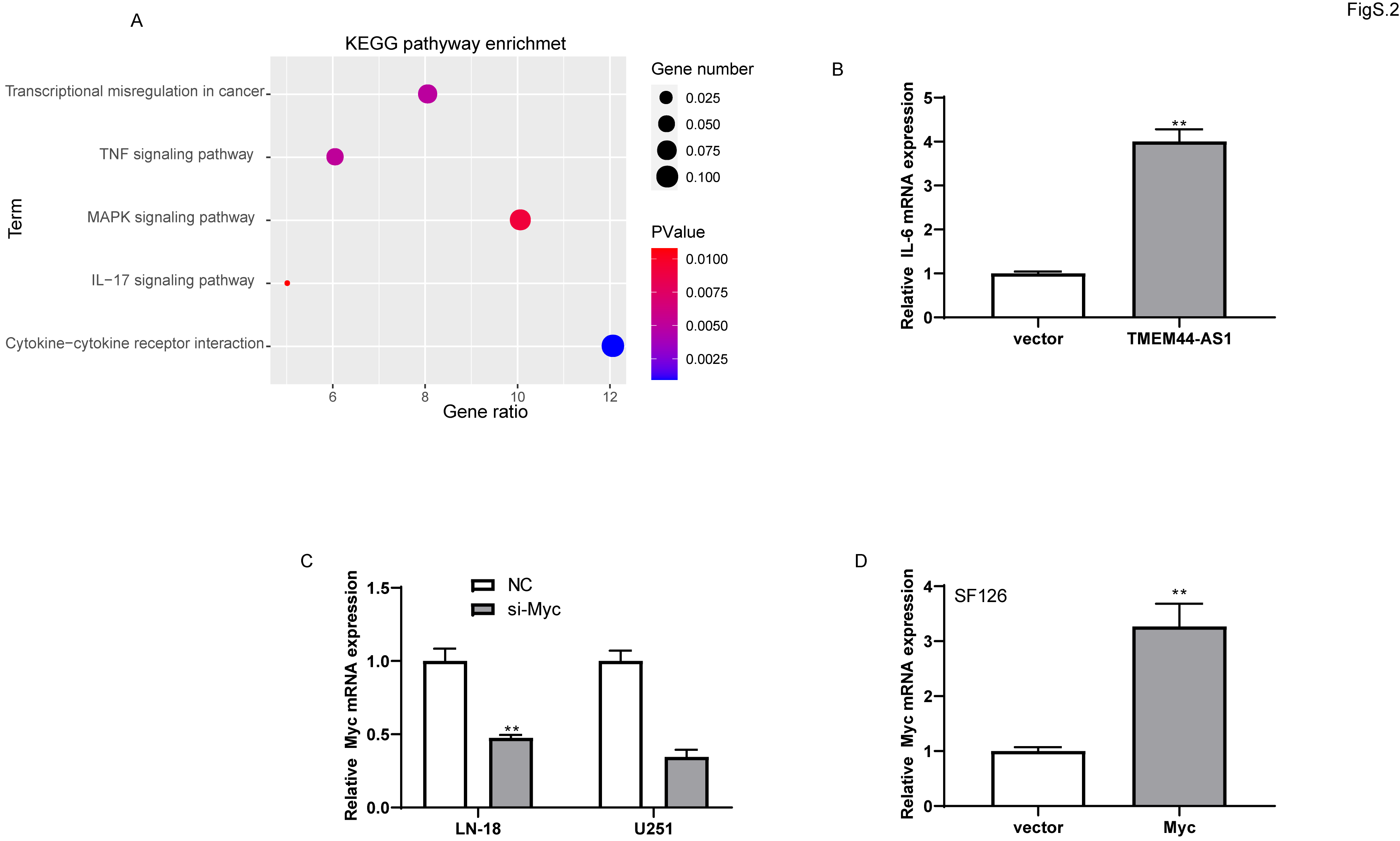

Supplement: Supplementary file 2 — Additional file 2:. [file 13046_2021_2129_MOESM2_ESM.tif]

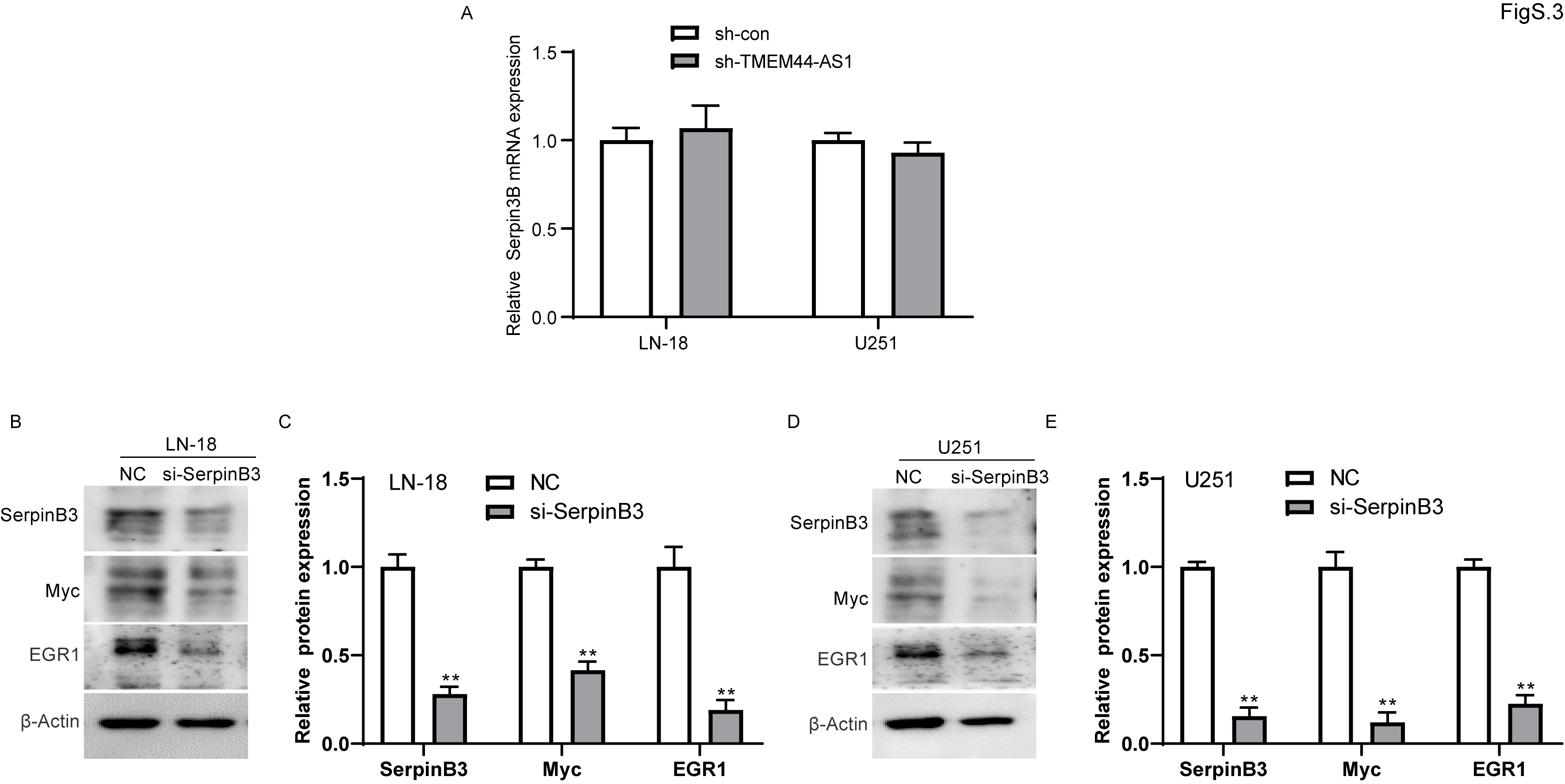

Supplement: Supplementary file 3 — Additional file 3:. [file 13046_2021_2129_MOESM3_ESM.tif]

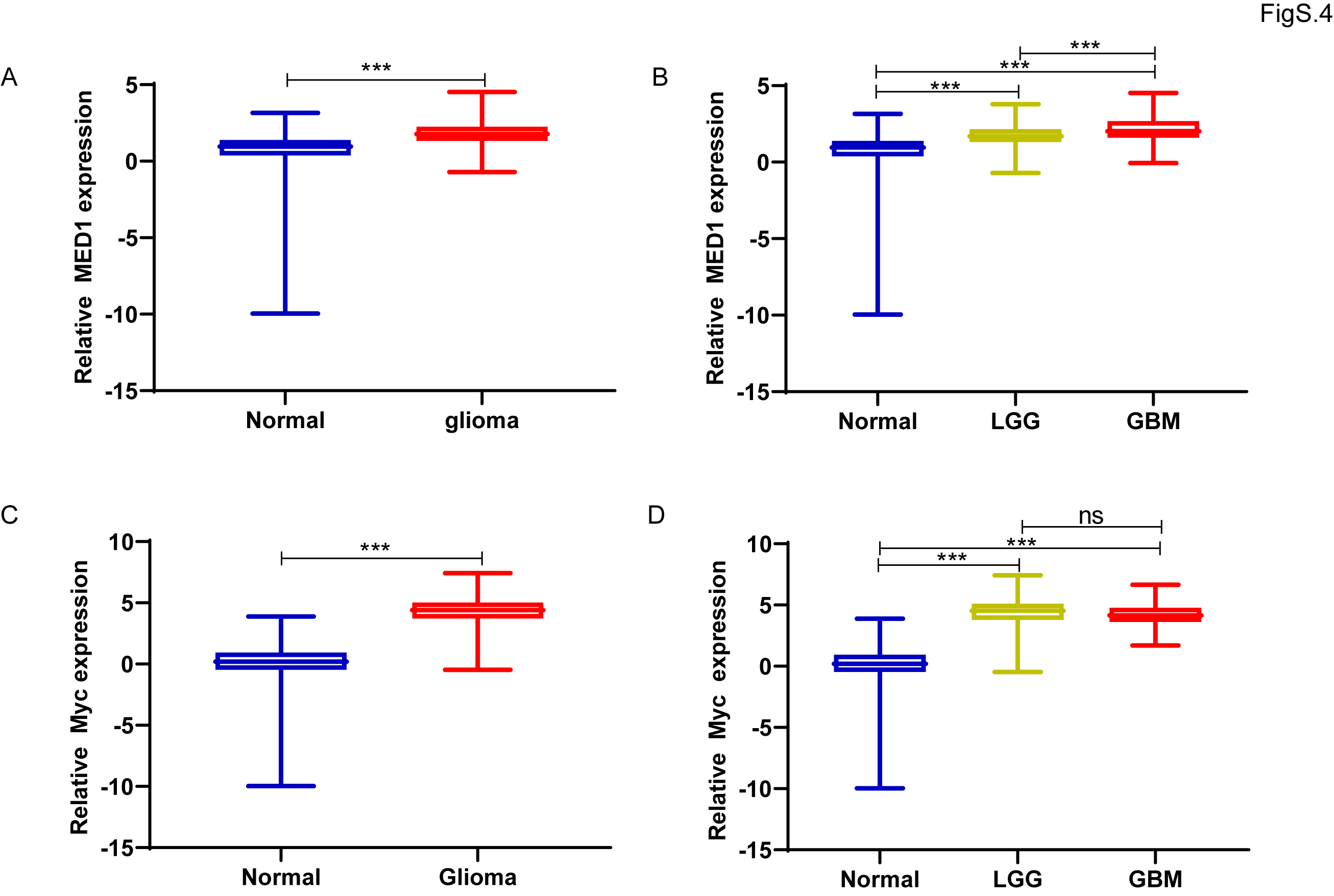

Supplement: Supplementary file 4 — Additional file 4:. [file 13046_2021_2129_MOESM4_ESM.tif]

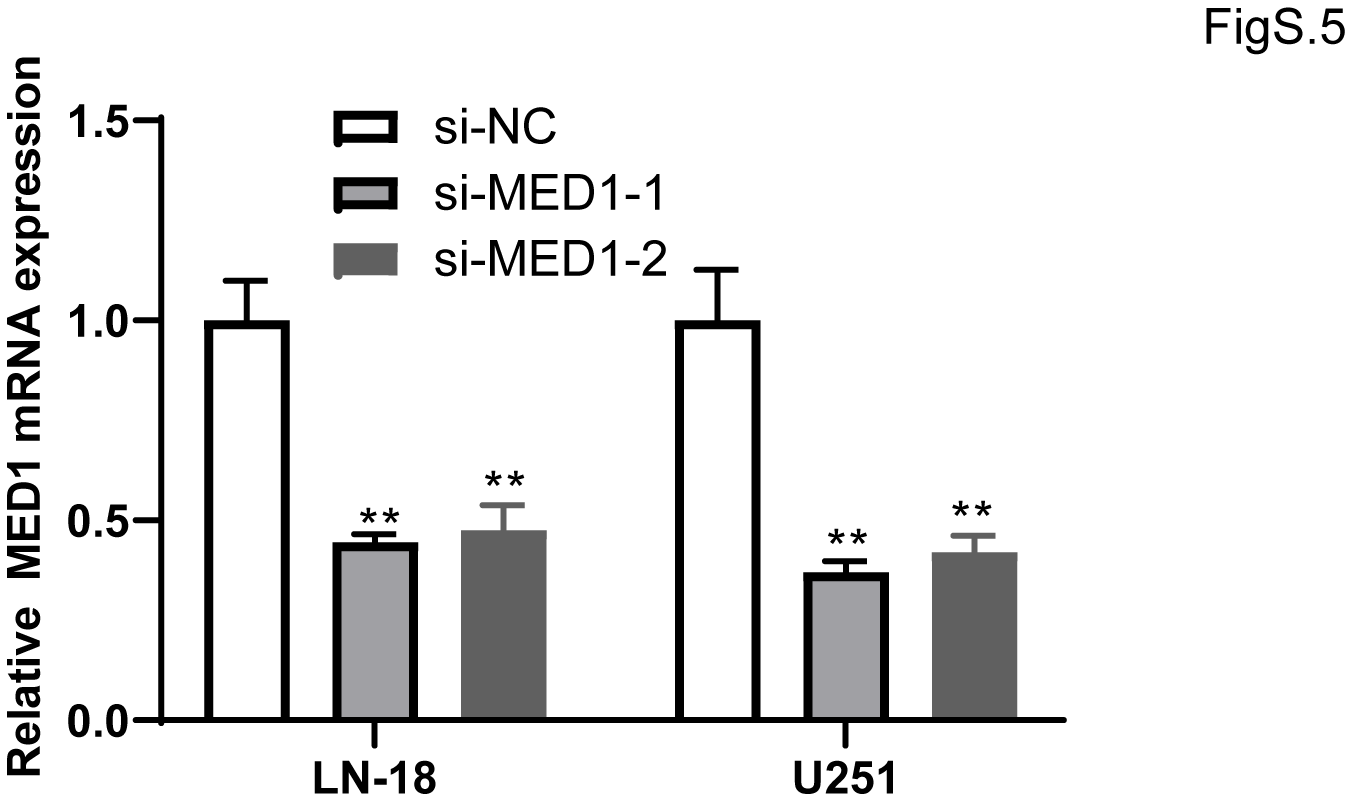

Supplement: Supplementary file 5 — Additional file 5:. [file 13046_2021_2129_MOESM5_ESM.tif]

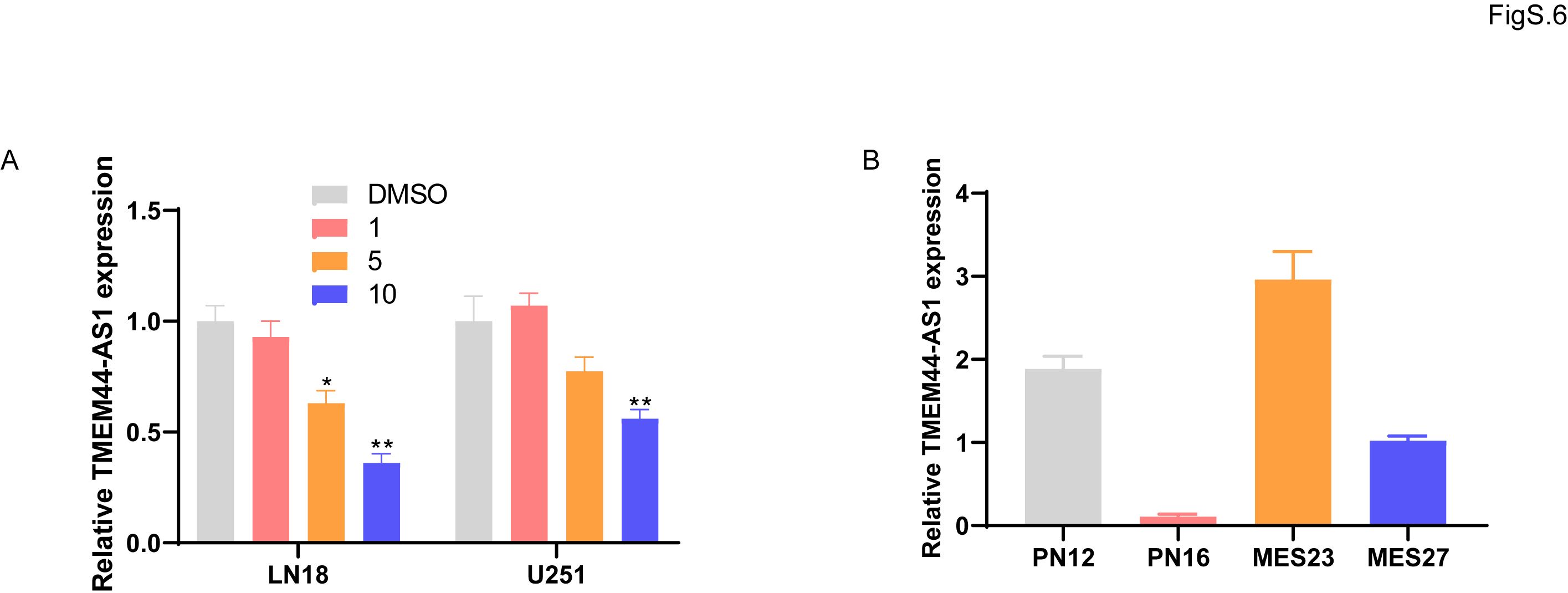

Supplement: Supplementary file 6 — Additional file 6:. [file 13046_2021_2129_MOESM6_ESM.tif]
