# Supplementary material for: Super-enhancer-associated TMEM44-AS1 aggravated glioma progression by forming a positive feedback loop with Myc
Source: J Exp Clin Cancer Res. 2021 Oct 25;40:337. doi: 10.1186/s13046-021-02129-9 (PMC8543865; doi:10.1186/s13046-021-02129-9)
Supplement: Supplementary file 7 — Additional file 7:. [file 13046_2021_2129_MOESM7_ESM.docx]

**Supplementary Table 1. Clinic-pathological characteristics of 46 glioma patients**

| Characteristic | n of patients |
| --- | --- |
|  |  |
| KPS |  |
| >80 | 35 |
| <80 | 11 |
| Gender |  |
| Male | 24 |
| Female | 22 |
| Age |  |
| ≤ 50 | 14 |
| > 50 | 32 |
| Grade |  |
| Low grade | 19 |
| GBM | 27 |

**Supplementary Table 2. Primers used for the construction of interference**

| Primer name | Sequence | |
| --- | --- | --- |
| Si-MED1-1 | 5’-GGAGGAAAGCUGAAACCAUTT-3’ | 5’-AUGGUUUCAGCUUUCCUCCTT-3’ |
| Si-MED1-2 | 5’-GCUCAUGAACCUUCUUAAATT-3’ | 5’-UUUAAGAAGGUUCAUGAGCTT-3’ |
| Si-TMEM44-AS1-1 | 5’-CCAAGCGUCUAUGAUGAUUTT-3’ | 5’-AAUCAUCAUAGACGCUUGGTT-3’ |
| Si-TMEM44-AS1-2 | 5’-CCAUUGACACAAUGGCCAUTT-3’ | 5’-AUGGCCAUUGUGUCAAUGGTT-3’ |
| Si-EGR1 | 5’-CAGUAUCAUCUCCAUCAUA-3’ | 5’-UAUGAUGGAGAUGAUACUG-3’ |
| Si-Myc-1 | 5’-GCUUGUACCUGCAGGAUCUTT-3’ | 5’-AGAUCCUGCAGGUACAAGCTT-3’ |
| Si-SerpinB3 | 5’-CCGCUGUAGUAGGGAUUCGGAU-3’ | 5’-AUCCGAAUCCCUACUACAGCGG-3’ |

**Supplementary Table 3. Primer pairs for promoter and enhancer regions in ChIP-qPCR**

|  | Forward Primer | Reverse Primer |
| --- | --- | --- |
| TMEM44-AS1-Pro | AGGCTGCAGTCAGCAGAGA | GGCGTCTCCGTAGTTCAGTT |
| TMEM44-AS1-SE1 | GCAACATAGCCAGACCCTGT | CCTTTCCACTCCCAAATGC |
| TMEM44-AS1-SE2 | TCGTGACAGTGTGGGTGAGT | GAGTGGCAGAGGCTGTTACC |
| TMEM44-AS1-SE3 | GTCACAGAGGGGATGTTGCT | AGAGTGCCTGGATGTTTGCT |
| TMEM44-AS1-SE4 | GGTGCTCTCAGACGCTTAGG | TGTGGGATGCTGTGTCAGA |
| TMEM44-AS1-SE5 | CTTTTCCCGTGCTCTCATGT | GGTCAGTGAGGAAAGCCTTG |
| IL6-pro | TTTCCAATCAGCCCCACC | GGAGTTCATAGCTGGGCTC |

**Supplementary Table 4. Primers used for RT-qPCR**

| **RT-qPCR** | | |
| --- | --- | --- |
| Primer name | Forward Primer | Reverse Primer |
| ABCB1 | AGGCCAACATACATGCCTTC | CCACCAGAGAGCTGAGTTCC |
| FOS | CTCCGGTGGTCACCTGTACT | GTCAGAGGAAGGCTCATTGC |
| IL-6 | GTTGTGCAAGGGTCTGGTTT | TCTTCCCTCAGGATGGTGTC |
| DDIT3 | TCTGATTGACCGAATGGTGA | TCTGGGAAAGGTGGGTAGTG |
| PLA2G4C | ACAATGACCTGCAACCTTCC | CTTCCGAAGTGGGTTATGGA |
| IL1A | AGCCTCAAGATGAAGGCAAA | GATGCCTGGTCACACTCAGA |
| DUSP6 | ATGGTAGTCCGCTGTCCAAC | ATTCCTCCAACACGTCCAAG |
| GADD45A | AACGGTGATGGCATCTGAAT | CCCTTGGCATCAGTTTCTGT |
| DUSP5 | AGGGTGCCTACTGCACATTC | CCTCCATCCCAGTTTTAGCA |
| EGR1 | CCGCAGAGTCTTTTCCTGAC | AGCGGCCAGTATAGGTGATG |
| Myc | ATTTGTGTCCCAAGCACTCC | GGGCATGTGGATGAGTCTTT |
| MED1 | GAGAATCCTGTGAGCTGTCCG | GTTGCTTTCCAGTACATAATTGC |
| TMEM44-AS1 | CCTGCTCCTACACGGTGTTT | ATGGGGAAACTGAGTCGGTG |

**Figure legends**

**FigS. 1** (A) Expression of TMEM44-AS1 in LN-18 and U251 cells was examined transfected with si-TMEM44-AS1 or si-NC. **P< 0.01 vs. si-NC. (B) Expression of TMEM44-AS1 in LN-18 and U251 cells was examined transfected with sh-TMEM44-AS1 or sh-con. **P< 0.01 vs. sh-con. (C) Expression of TMEM44-AS1 in SF126 cells was examined transfected with TMEM44-AS1 plasmid or vector. **P< 0.01 vs. vector.

**FigS. 2**

1. KEGG pathways associated with genes differentially expressed upon TMEM44-AS1 knockdown in LN-18 glioma cells. (B) Expression of IL-6 in SF126 cells was examined transfected with vector or TMEM44-AS1 plasmid. **P< 0.01. (C) Expression of Myc in LN-18 and U251 cells was examined transfected with si-Myc or si-NC. **P< 0.01 vs. si-NC. (D) Expression of TMEM44-AS1 in SF126 cells was examined transfected with Myc plasmid or vector. **P< 0.01 vs. vector.

**FigS. 3**

(A) RT-qPCR assays measuring the expression of SerpinB3 upon knockdown of TMEM44-AS1 in LN-18 and U251 cells. (B-E) Western blot assays measuring the expression of Myc, EGR1 and SerpinB3 protein upon knockdown of SerpinB3 in LN-18 and U251 cells. **P< 0.01.

**FigS. 4**

(A) Relative MED1 expression in glioma and normal brain tissues from GTEX (n=1136) and TCGA database (n=689). ***P< 0.001 vs. normal. (B) The MED1 expression is shown according to the histopathologic grades of TCGA gliomas. LGG (n=523); GBM (n=166). ***P< 0.001 vs. normal, ***P< 0.001 vs. LGG. (C) Relative Myc expression in glioma and normal brain tissues, ***P< 0.001 vs. normal. (D) The Myc expression is shown according to the histopathologic grades of TCGA gliomas. ***P< 0.001 vs. normal.

**FigS. 5**

Expression of MED1 was examined in LN-18 and U251 cells transfected with si- MED1 or si-NC. **P< 0.01 vs. si-NC.

**FigS. 6**

(A) Expression of TMEM44-AS was examined in LN-18 and U251 cells treated with DMSO or Myci975 at an indicated concentration. *P< 0.05, **P< 0.01 vs. DMSO. (B) Expression of TMEM44-AS1 in patient-derived glioblastoma cells.
